# Significant role of long non-coding RNA MALAT1 in deep vein thrombosis via the regulation of vascular endothelial cell physiology through the microRNA-383-5p/BCL2L11 axis

**DOI:** 10.1080/21655979.2022.2080412

**Published:** 2022-06-15

**Authors:** Hecheng Wang, Shusen Lin, Yujie Yang, Mingyu Zhao, Xichun Li, Lanli Zhang

**Affiliations:** a Department of Academic Affairs, The Third Affiliated Hospital of Qiqihar Medical College, Qiqihar 161000, China; b Department of Vascular Surgery, The Third Affiliated Hospital of Qiqihar Medical College, Qiqihar 161000, China; c Department of Ultrasound, The Third Affiliated Hospital of Qiqihar Medical College, Qiqihar 161000, Chin

**Keywords:** lncRNA-MALAT1, miR-383-5p, BCL2L11, HUVECs, deep vein thrombosis

## Abstract

Deep vein thrombosis (DVT) is a vascular disease. The long non-coding RNA (lncRNA), metastasis-associated lung adenocarcinoma transcript 1 (MALAT1), is positively expressed in DVT tissues, and regulates the biological behavior of endothelial progenitor cells. Here, we explored whether MALAT1 affected the physiology of human vascular endothelial cells (HUVECs) and analyzed its underlying mechanism. To overexpress/silence the expression of MALAT1 in HUVECs, MALAT1-plasmid/MALAT1-small interfering RNA (siRNA) was used. The 3-(4,5-dimethylthiazol-2-yl)-2,5-diphenyl tetrazolium bromide and flow cytometry analyses were performed to observe the cell viability and apoptosis. Reverse transcription-quantitative polymerase chain reaction and western blotting were used to determine the apoptosis-related protein and gene expression levels. We used Starbase software to predict the associations among MALAT1, microRNA (miR)-383-5p, and BCL2-like 11 (BCL2L11). Luciferase reporter assay was used to validate their relationship. Compared to the control vector group, MALAT1-plasmid suppressed the viability and induced apoptosis of HUVECs, while improving Bcl-2-associated X protein (Bax) expression and decreasing Bcl-2 expression. There was an interaction between MALAT1 and miR-383-5p. Compared to the control siRNA group, MALAT1-siRNA increased the cell viability, reduced cell apoptosis, upregulated Bcl-2 expression, and suppressed Bax expression. These changes were reversed by the miR-383-5p inhibitor. Additionally, we verified that BCL2L11 is a target of miR-383-5p. miR-383-5p improved the cell proliferation, while decreasing cell apoptosis in HUVECs by targeting BCL2L11. Therefore, the lncRNA-MALAT1/miR-383-5p/BCL2L11 axis may be effective for DVT treatment.

## Graphical Abstract

**Figure:**
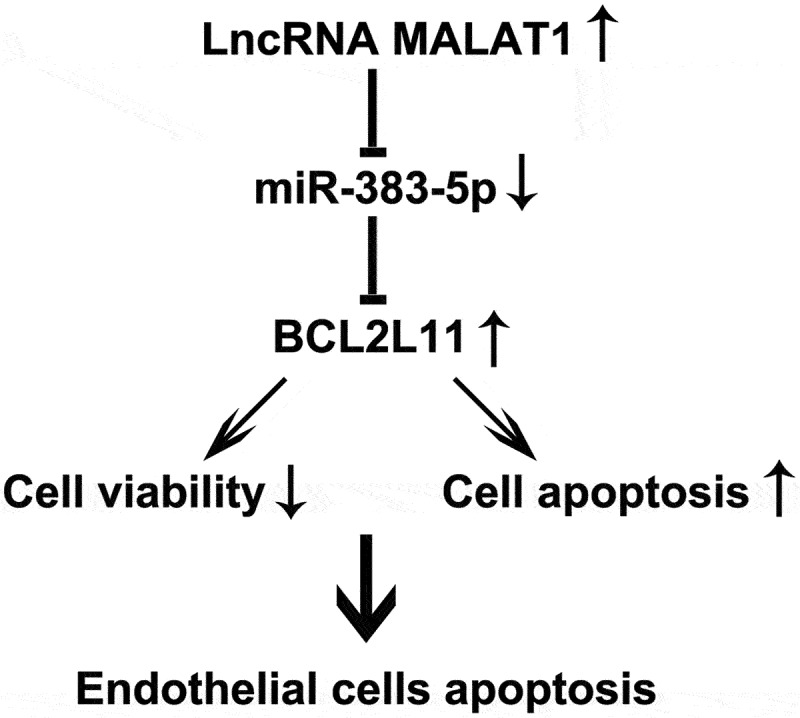

## Highlights

- MALAT1-plasmid significantly induced the apoptosis of HUVECs;
- miR-383-5p inhibitor reversed the effects of MALAT1-siRNA on HUVECs;
- BCL2L11 reversed the effects of miR-383-5p on HUVECs.

## Introduction

Deep vein thrombosis (DVT), which refers to the aberrant coagulation of blood in deep veins to form a blood clot, mainly occurs in the veins of the legs [1]. Lower-extremity DVT is a common complication in trauma patients, with a high incidence and mortality rate [2]. Thrombosis leads to impaired pulmonary circulation and respiratory function, which can cause pulmonary embolism and sudden death of the patient [3,4]. With increasing age, the incidence of DVT is also increases [5], and male patients show a higher incidence than female patients [6]. However, the pathogenesis of DVT requires further investigation. At present, patients are mainly diagnosed based on their clinical symptoms and imaging findings. Therefore, we aimed to establish a biomarker for the early prediction, diagnosis, and treatment of DVT.

Aging of vascular endothelial cells (ECs) and reduction in anticoagulant and fibrinolytic activities accelerate the DVT process [7–9]. Damage to the blood vessel wall can easily trigger venous thrombosis [10,11]. Vascular endothelial cells are associated with the development of DVT [12,13]. Cellular apoptosis of ECs greatly reduces their ability to release active substances, weakens their multiple defense functions in blood vessels, and prevents anticoagulation, and eventually facilitates DVT [14–18].

Long non-coding RNAs (lncRNAs) consist of approximately 100 base pairs; they are non-protein-coding RNAs and can only perform their biological functions at the RNA level [19]. LncRNAs are involved in many crucial biological processes that regulate the epigenetic expression, cell cycle, and cell differentiation [20,21]. Emerging evidence has confirmed the therapeutic effects of lncRNAs in DVT [22]. The lncRNA, metastasis-associated lung adenocarcinoma transcript 1 (MALAT1), was initially discovered in non-small cell lung cancer and was associated with lung cancer metastasis [23]. MALAT1 expression is abnormal in multiple cancers and is involved in cancer metastasis and development [24–26]. Gao et al. demonstrated that MALAT1 is involved in ischemic stroke development and that its expression is downregulated in brain microvascular endothelial cells under oxygen-glucose deprivation conditions [27]. Theo et al. showed that MALAT1 is aberrantly expressed in patients with Parkinson’s disease [28]. Furthermore, Du et al. showed that MALAT1 is positively expressed in DVT tissues. MALAT1 can regulate the biological behavior of endothelial progenitor cells, including cell proliferation, migration, cell cycle arrest, and cellular apoptosis [29]. The purpose of this study was to explore whether MALAT1 affects the physiology of ECs and to analyze its potential molecular mechanism.

MicroRNAs (miRNAs) consist of approximately 20–22 nucleotides, in contrast to protein-coding mRNAs. miRNAs are non-protein-coding and can inhibit the expression of their target genes [30–32]. miR-383-5p is present on chromosome 8 in humans. miR-383-5p acts as a tumor suppressor that is negatively regulated in many cancers, including ovarian, cervical, and gastric cancers [33–35]. However, the role of miR-383-5p in DVT remains unknown. It is worth mentioning that through the analysis of bioinformatics software, we found that there may be a certain interaction between MALAT1 and miR-383-5p.

In this study, we hypothesized that MALAT1 affects the physiology of ECs through regulating miR-383-5p expression in DVT. Therefore, this study explored the potential roles of MALAT1 and miR-383-5p in DVT.

## Materials and methods

### Reverse transcription-quantitative polymerase chain reaction (RT-qPCR) assay

Total RNA was extracted using the TRIzol reagent (TaKara, Shiga, Japan), according to the manufacturer’s instructions. RNA concentration was detected using a NanoDrop spectrophotometer (Thermo Scientific, USA). After extraction, the RNA (1 μg) was transformed into cDNA using the PrimeScript RT Reagent Kit (TaKara). Subsequently, qPCR was performed using the SYBR Green PCR kit (Vazyme, Nanjing, Jiangsu), according to the manufacturer’s instructions. The thermocycling conditions were as follows: Initial denaturation at 95°C for 15 min; followed by 40 cycles at 95°C for 15 sec (denaturation), 60°C for 15 sec (annealing) and 72°C for 15 sec (extension). U6 (miRNA) and GAPDH (mRNA) were used as the internal controls. Gene expression was calculated using the 2^−ΔΔCt^ formula [36]. Primer sequences for PCR were listed as following:

GAPDH forward, 5’-CATCATCCCTGCCTCTACTGG-3’;

reverse, 5’-GTGGGTGTCGCTGTTGAAGTC-3’;

U6 S, 5’-GGAACGATACAGAGAAGATTAGC-3’;

Stem-loop-R, 5’-CTCAACTGGTGTCGTGGAGTC-3’;

MALAT1 forward, 5ʹ-ATACCTAACCAGGCATAACA-3ʹ;

reverse, 5ʹ-AGTAGACCAACTAAGCGAAT-3ʹ;

miR-383-5p forward, 5’-GGGAGATCAGAAGGTGATTGTGGCT-3’;

reverse, 5ʹ-CAGTGCGTGTCGTGGAGT-3’;

BCL2L11 forward, 5ʹ-TAAGTTCTGAGTGTGACCGAGA-3’;

reverse, 5ʹ-GCTCTGTCTGTAGGGAGGTAGG-3’;

Bcl-2 forward, 5ʹ-GGTGGGGTCATGTGTGTGG-3’;

reverse, 5ʹ-CGGTTCAGGTACTCAGTCATCC-3’;

Baxforward, 5ʹ-CCCGAGAGGTCTTTTTCCGAG-3’;

reverse, 5ʹ-CCAGCCCATGATGGTTCTGAT-3’.

### 3-(4,5-dimethylthiazol-2-yl)-2,5-diphenyltetrazolium bromide (MTT) assay

Transfected cells were seeded into a 96-well microplate, and incubated for 24 h. Then, 20 µL of MTT (5 mg/mL; Sigma, St. Louis, MO, USA) was added to each well and the cells were cultured for an additional 4 h. Subsequently, the supernatant was discarded and 200 µL of dimethyl sulfoxide was added to the wells. The absorbance of the samples was measured at 570 nm wavelength using a microplate reader [37].

### Flow cytometry (FCM) assay

Transfected cells were analyzed using the Annexin V/propidium iodide (PI) Apoptosis Detection Kit (Beyotime, Shanghai, China) [38]. In brief, we washed the cells twice with pre-cooled 1x phosphate-buffered saline before collecting the cells, and then prepared a cell suspension of 1 × 10^6^ cells/mL using the fluorescein isothiocyanate (FITC)-binding buffer. We added 100 μL of the cell suspension to EP tubes. Subsequently, we added an appropriate amount of Annexin V-FITC and PI to the cells, according to the standard operating protocol. Cells were gently mixed and incubated for 20 min at 25°C in the dark. Cell apoptosis were analyzed using a BD FACSCalibur flow cytometer (BD Technologies). The data were analyzed using Kaluza Analysis (version 2.1.1.20653; Beckman Coulter, Inc.).

### Western blotting analysis

Protein expression levels were determined using western blot assay [39]. To extract the total proteins, the cells were processed with the radioimmunoprecipitation assay buffer (Solarbio, Beijing, China). The protein concentration was measured using the bicinchoninic acid assay kit (Pierce, Appleton, WI, USA). Sodium dodecyl sulfate-polyacrylamide gel (12%) was used for protein isolation, which were subsequently transferred to PVDF membranes. Nonfat milk (5%) was used to block the membranes to avoid nonspecific binding and incubated with primary antibodies, including anti-Bcl-2-associated X protein (Bax) (cat. no. ab32503; 1:1000; Abcam, Cambridge, MA, USA), anti-Bcl-2 (cat. no. ab32124; 1:1000; Abcam, Cambridge, MA, USA), BCL2L11 (cat. no. ab32158; 1:1000; Abcam, Cambridge, MA, USA), and GAPDH (cat. no. ab9485; 1:1000; Abcam, Cambridge, MA, USA) antibodies, at 4°C overnight. After incubation for 24 h, the membranes were incubated with the secondary antibody (cat. no. ab7090; 1:1000; Abcam, Cambridge, MA, USA) for 2 h. Eventually, the ECL method (Applygen Technologies, Inc., Beijing, China) was used to visualize and measure the protein signals.

### Dual luciferase reporter analysis

StarBase database (http://starbase.sysu.edu.cn/) was used to predict the binding site of lncRNA MALAT1 and miR-383-5p, and the binding sites between miR-383-5p and BCL2L11.

To validate the interaction between MALAT1 and miR-383-5p, the wild-type (WT) or mutant (MUT) MALAT1 was cloned into the pmiRGLO vector (Promega, Madison, WI, USA) following the manufacturer’s instructions. They were co-transfected with miR-383-5p and Renilla luciferase into 293 T cells. QuikChange Site-Directed Mutagenesis kit (Stratagene; Agilent Technologies, Inc.) was used to point-mutate the miR-383-5p binding domain in MALAT1 as per the manufacturer’s instructions. After 48 h of transfection, luciferase activity was measured using a dual-luciferase reporter assay system (Promega, Madison, WI, USA) [40].

To investigate the reaction of miR-383-5p and BCL2-like 11 (BCL2L11), WT-BCL2L11 and MUT-BCL2L11 3ʹ-untranslated region (UTR) luciferase reporter gene vectors were used for transfection. QuikChange Site-Directed Mutagenesis kit (Stratagene; Agilent Technologies, Inc.) was used to point-mutate the miR-383-5p binding domain in the 3ʹUTR of BCL2L11 according to the manufacturer’s instructions. The cells were transfected with Renilla luciferase, luciferase reporter gene plasmids, and miR-383-5p mimic or mimic control for 48 h. Luciferase activity was measured using a dual-luciferase reporter assay system, according to the manufacturer’s instructions.

### Cell culture and transfection

Human vascular endothelial cells (HUVECs) were obtained from the American Tissue Culture Collection (ATCC, Manassas, VA, USA). The cells were cultured in Dulbecco’s modified Eagle’s medium (Gibco, NY, USA) containing 10% fetal bovine serum FBS (Gibco) at 37°C in a 5% CO_2_ incubator.

To overexpress/silence the expression of MALAT1 in HUVECs, MALAT1-plasmid/MALAT1-small interfering RNA (siRNA) (Guangzhou RiboBio Co., Ltd., Guangzhou, China) was used, and the control-plasmid/control-siRNA was used as the negatively control (Guangzhou RiboBio Co., Ltd., Guangzhou, China); To down-regulate/up-regulate the level of miR-383-3p in HUVECs, miR-383-3p inhibitor/miR-383-3p mimic (Guangzhou RiboBio Co., Ltd., Guangzhou, China) was used, and the inhibitor control/mimic control was used as the negatively control (Guangzhou RiboBio Co., Ltd., Guangzhou, China); To overexpress BCL2L11 expression in HUVECs, BCL2L11-plasmid (Santa Cruz Biotechnology; Santa Cruz, CA, USA) was used, and control-plasmid (Santa Cruz Biotechnology; Santa Cruz, CA, USA) was used as the negative control.

HUVECs were transfected with MALAT1-plasmid, MALAT1-siRNA, miR-383-3p inhibitor, MALAT1-siRNA + inhibitor control, MALAT1-siRNA + miR-383-3p inhibitor, miR-383-3p mimic, miR-383-3p mimic + control-plasmid, or miR-383-3p mimic + BCL2L11-plasmid using the Lipofectamine™3000 (Invitrogen, Carlsbad, CA, USA) for 48 h, following the manufacturer’s instructions. Cells without any treatment were considered as control. After 48 h, the transfected cells were harvested for further experiments.

### Statistical analysis

SPSS 11 software (IBM, Armonk, NY, USA) was used for data analysis. Statistical significance of the differences between two groups was determined using the Student’s *t*-test, and the differences between multiple groups was analyzed by one-way ANOVA followed by post hoc Tukey’s test. The results are presented as the mean ± standard error of three independent experiments. Statistical significance was set at p < 0.05.

## Results

### Effects of lncRNA MALAT1 on the proliferation and apoptosis of HUVECs

**Figure 1.**
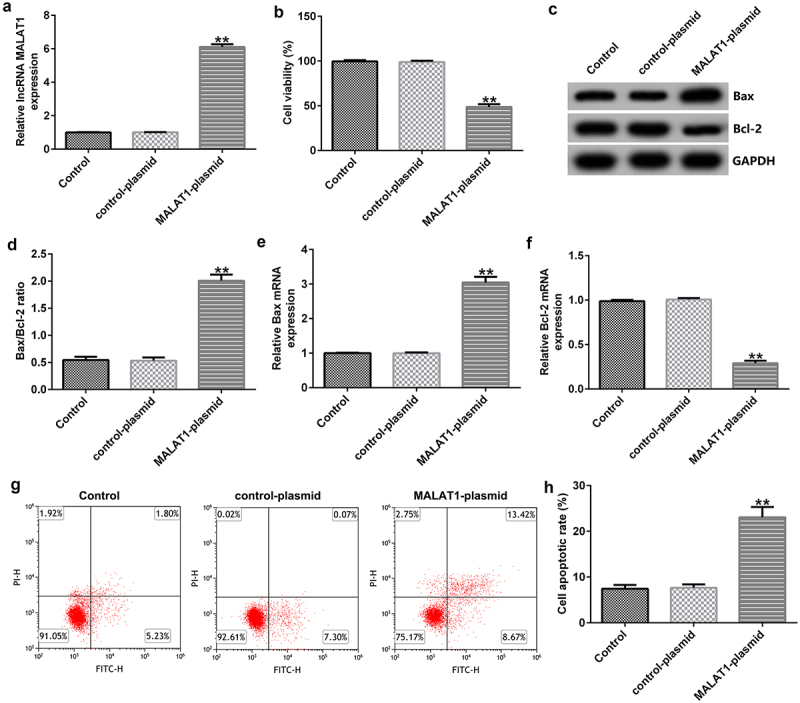
Upregulation of metastasis-associated lung adenocarcinoma transcript 1 (MALAT1) expression suppresses cell proliferation. (a) Quantitative reverse transcription-polymerase chain reaction (qRT-PCR) assay was used to determined MALAT1 expression levels when human vascular endothelial cells (HUVECs) were transfected with the control-plasmid or MALAT1-plasmid for 48 h. (b) 3-(4,5-dimethylthiazol-2-yl)-2,5-diphenyl tetrazolium bromide (MTT) assay of cell viability. (c) Bcl-2-associated X (Bax) and Bcl-2 protein expression levels were determined via western blotting analysis. (d) The ratio of Bax/Bcl-2 is shown. qRT-PCR assay was used to measure Bax (e) and Bcl-2 (f) mRNA expression levels. (g) Flow cytometry (FCM) assay was performed to detect cell apoptosis. (h) Apoptosis rate of HUVECs.

To determine the influence of MALAT1 on HUVEC viability, control-plasmid (the negative control) or MALAT1-plasmid (to overexpress MALAT1 expression) was transfected into HUVECs. 48 h after cell transfection, qRT-PCR analysis was used to determine the expression of MALAT1, and the results showed that the MALAT1-plasmid improved MALAT1 expression levels in HUVECs compared with the control plasmid group (figure 1(a)). We then performed MTT and FCM assays to measure the cell viability and apoptosis. Our results indicated that, compared to the control plasmid group, the MALAT1-plasmid significantly inhibited the cell viability (figure 1(b)). Western blotting and RT-qPCR analyses indicated that MALAT1-plasmid upregulated Bax protein and mRNA expression levels, downregulated Bcl-2 protein and mRNA expression levels, and upregulated the ratio of Bax/Bcl-2 in HUVECs (figure 1(c–f)). In addition, compared to the control plasmid group, MALAT1-plasmid significantly induced the apoptosis of HUVECs (figure 1(g,h)).

### Determination of the binding sites of lncRNA MALAT1 and miR-383-5p

**Figure 2.**
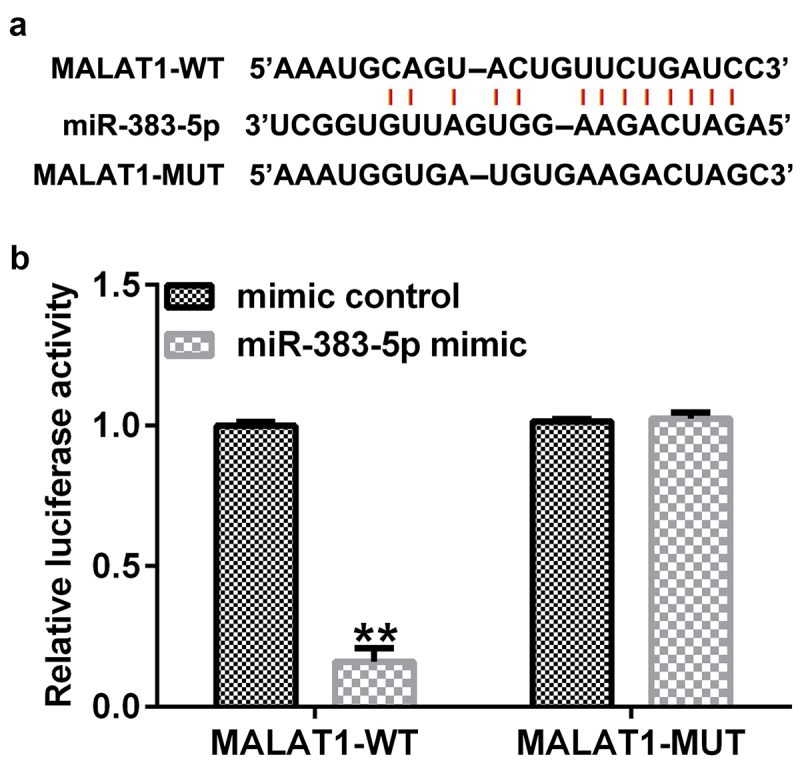
Binding of long non-coding RNA (lncRNA) MALAT1 with microRNA (miR)-383-5p. (a) Interaction between miR-383-5p and 3ʹ-untranslated region (UTR) of MALAT1 was predicted using the starbase prediction software. (b) Dual luciferase reporter gene assay was used to verify the interaction between MALAT1 and miR-383-5p when 293 T cells were co-transfected with the miR-383-5p mimic and wild-type or mutant MALAT1 3ʹ-UTR reporter.

To predict the relationship between lncRNA MALAT1 and miR-383-5p, Starbase analysis was used, and the results indicated that there were interaction sites between lncRNA MALAT1 and miR-383-5p (figure 2(a)). To validate the association between MALAT1 and miR-383-5p, we performed a dual-luciferase reporter assay. The cells (293 T) were transfected with MALAT1-WT or MALAT1-MUT luciferase reporter plasmid, miR-383-5p mimic, or Renilla luciferase reporter plasmid for 48 h using the Lipofectamine 3000 reagent. The dual-luciferase reporter assay indicated that the miR-383-5p mimic could inhibit the activity of MALAT1-WT, but not MALAT1-MUT (figure 2(b)).

### MALAT1 negatively regulates miR-383-5p expression levels in HUVECs

**Figure 3.**
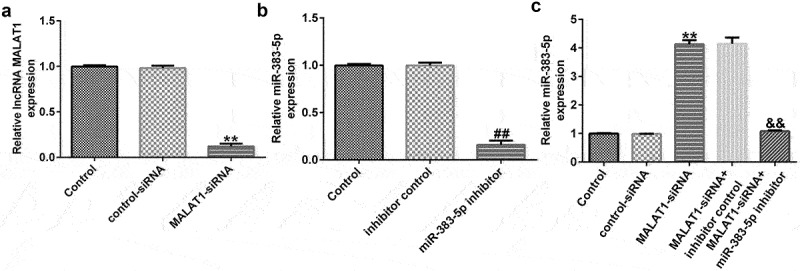
MALAT1 negatively regulates miR-383-5p expression levels in HUVECs. (a). qRT-PCR assay was used to determine MALAT1 expression levels when HUVECs were transfected with the control-small interfering RNA (siRNA) or MALAT1-siRNA. (b) qRT-PCR assay was used to determine miR-383-5p expression levels when HUVECs were transfected with the inhibitor control or miR-383-5p inhibitor. (c) qRT-PCR assay was used to determine miR-383-5p expression levels when HUVECs were transfected with the control-siRNA, MALAT1-siRNA, MALAT1-siRNA + inhibitor control or MALAT1-siRNA + miR-383-5p inhibitor for 48 h.

To explore the potential mechanism by which MALAT1 and miR-383-5p affect the proliferation and apoptosis of HUVECs, HUVECs were transfected with the control-siRNA, MALAT1-siRNA, inhibitor control, miR-383-5p inhibitor, MALAT1-siRNA + inhibitor control, or MALAT1-siRNA + miR-383-5p inhibitor for 48 h. qRT-PCR analysis showed that MALAT1-siRNA significantly decreased MALAT1 expression levels compared to the control siRNA (figure 3(a)). The miR-383-5p inhibitor reduced miR-383-5p expression levels compared to the inhibitor control group (figure 3(b)). Compared to the control siRNA group, MALAT1-siRNA significantly increased miR-383-5p expression levels in HUVECs, and this effect was reversed by the miR-383-5p inhibitor (figure 3(c)).

### Effects of MALAT1-siRNA and miR-383-5p on the activity of HUVECs

**Figure 4.**
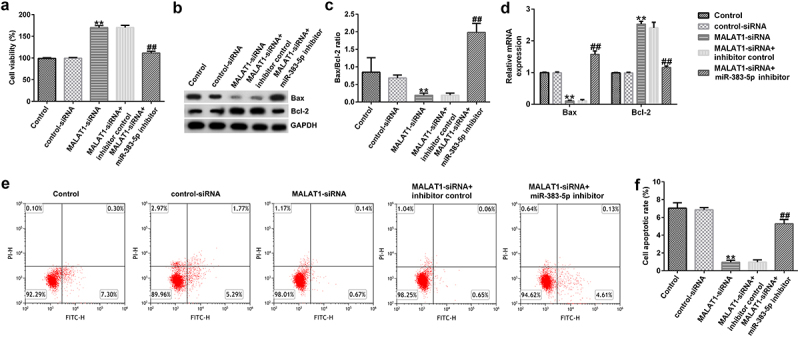
MALAT1-siRNA increases the viability and inhibits the apoptosis of HUVECs by upregulating miR-383-5p expression levels. (a) MTT assay to determine cell viability. (b) Bax and Bcl-2 protein expression levels were determined via western blotting analysis. (c) The ratio of Bax/Bcl-2 is shown. (d) qRT-PCR assay was used to determine Bax and Bcl-2 mRNA expression levels. (e) FCM assay was performed to detect cell apoptosis. (f) Apoptosis rate of HUVECs.

Next, to explore the effect of low MALAT1 expression on HUVEC viability and apoptosis, MTT and FCM assays were performed. Our data indicated that, compared to the control siRNA group, MALAT1-siRNA increased the viability of HUVECs (figure 4(a)). qRT-PCR and western blotting assays indicated that MALAT1-siRNA decreased Bax protein and mRNA expression levels, increased Bcl-2 expression levels, and reduced the ratio of Bax/Bcl-2 (figure 4(b–d)). Moreover, compared to the control siRNA group, MALAT1-siRNA significantly reduced cell apoptosis (figure 4(e,f)). These changes were neutralized by the miR-383-5p inhibitor.

### BCL2L11 *is a direct target gene of miR-383-5p*

**Figure 5.**
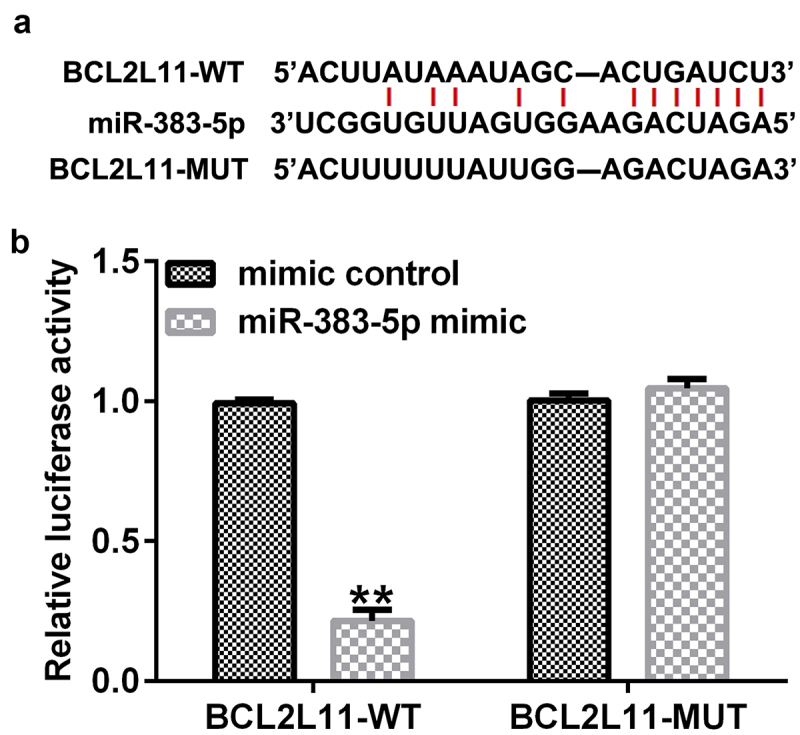
Targeting relationship between miR-383-5p and BCL2-like 11 (BCL2L11). (a) Starbase analysis was used to predict the relationship between miR-383-5p and BCL2L11. (b) Binding relationship of miR-383-5p and BCL2L11 was confirmed using the dual-luciferase reporter assay.

We then performed Starbase analysis to predict the presence of a binding site for miR-383-5p and BCL2L11 (figure 5(a)). A dual-luciferase reporter assay was performed to validate these results. The cells (293 T) were transfected with BCL2L11-WT or BCL2L11-MUT luciferase reporter plasmid, miR-383-5p mimic, and Renilla luciferase reporter plasmid for 48 h. The dual-luciferase reporter assay showed that the miR-383-5p mimic inhibited the activity of BCL2L11-WT, but not BCL2L11-MUT (figure 5(b)). Altogether, *BCL2L11* was determined to be a target gene of miR-383-5p.

### miR-383-5p negatively regulates BCL2L11 expression levels in HUVECs

**Figure 6.**
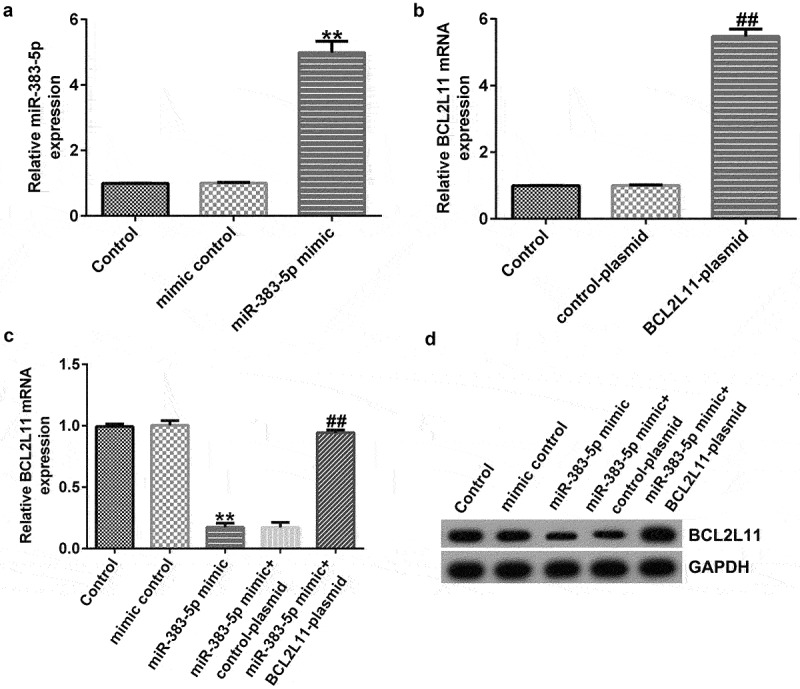
miR-383-5p negatively regulates BCL2L11 expression levels in HUVECs. (a) qRT-PCR assay was used to determine miR-383-5p expression levels when HUVECs were transfected with the miR-383-5p mimic. (b) qRT-PCR assay was used to determine BCL2L11 expression levels when HUVECs were transfected with the BCL2L11-plasmid. (c) qRT-PCR assay was used to determine BCL2L11 expression levels when HUVECs were transfected with the mimic control, miR-383-5p mimic, miR-383-5p mimic + control-plasmid, or miR-383-5p mimic + BCL2L11-plasmid. (d) BCL2L11 expression levels were determine using western blot analysis.

To explore the biological activity of miR-383-5p and BCL2L11 in HUVECs, HUVECs were transfected with the miR-383-5p mimic, BCL2L11-plasmid, or miR-383-5p mimic + BCL2L11-plasmid for 48 h. qRT-PCR and western blotting were performed to measure the efficiency of cellular transfection. Compared to the mimic control group, the miR-383-5p mimic promoted miR-383-5p expression levels (figure 6(a)). Compared to the control plasmid group, the BCL2L11-plasmid increased BCL2L11 mRNA expression levels (figure 6(b)). In addition, compared to the mimic control group, the miR-383-5p mimic reduced BCL2L11 mRNA and protein expression levels in HUVECs, but this effect was reversed by the BCL2L11-plasmid (figure 6(c,d)).

### Effects of miR-383-5p and BCL2L11 on the proliferation and apoptosis of HUVECs

**Figure 7.**
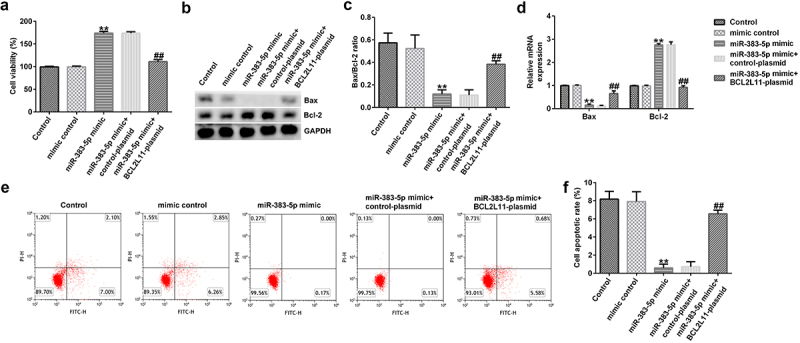
miR-383-5p improves the viability and decreases the apoptosis of cells by downregulating BCL2L11 expression levels. (a) MTT assay to detect cell viability. (b) Bax and Bcl-2 protein expression levels were determined via western blotting analysis. (c) The ratio of Bax/Bcl-2 is shown. (d) qRT-PCR assay was used to determine Bax and Bcl-2 mRNA expression levels. (e) FCM assay was performed to detect cell apoptosis. (f) Apoptosis rate of HUVECs.

To investigate the effects of miR-383-5p and BCL2L11 on the activity of HUVECs, we conducted experiments with reference to previous research methods. MTT analysis indicated that the miR-383-5p mimic significantly promoted cell proliferation (figure 7(a)). The miR-383-5p mimic also upregulated Bax protein and mRNA and Bcl-2 expression levels, while reducing the ratio of Bax/Bcl-2 (figure 7(b–d)). Furthermore, the miR-383-5p mimic decreased cell apoptosis (figure 7(e,f)). These changes were eliminated via BCL2L11-plasmid co-transfection. These results suggest that miR-383-5p improves cell viability by downregulating BCL2L11 expression.

## Discussion

Thrombosis is a difficult problem in clinical practice. Thrombosis is a vascular disease, with an incidence of approximately 0.1% worldwide [41]. DVT can cause leg swelling, ulcers, post-thrombotic syndrome, and pulmonary embolism, which can result in the death of approximately 15% of patients within the first three months of diagnosis [41]. Anticoagulants are now widely used to treat DVT; however, some complications still affect the quality of life of the patients with DVT [42]. Therefore, development of targeted therapy is necessary to treat DVT.

In recent years, studies have found that lncRNAs play an important role in the occurrence and development of DVT [22,43]. MALAT1 is also involved in DVT development [29]. Damage to vascular endothelial cells plays an important role in the occurrence of DVT [10,11]. In this study, our findings showed that MALAT1 suppressed the viability, while inducing the apoptosis of HUVECs. Our results are similar to those reported by Du et al. Du et al. showed that MALAT1 inhibited EPC proliferation [29].

An increasing number of studies have shown interactions between lncRNAs and miRNAs. LncRNAs can directly interact with miRNAs as competitive endogenous RNAs, thereby regulating their expression levels and activities [44]. Lv et al. revealed that miR-135b-5p is a target of MALAT1, and MALAT1 regulates the cell viability by regulating miR-135b-5p expression in Parkinson’s disease [45]. Jia et al. showed that MALAT1 is involved in ischemia-reperfusion injury via miR-195a-5p [46]. Our results revealed the target relationship between lncRNA MALAT1 and miR-383-5p in HUVECs, and low MALAT1 expression improved the viability and decreased the apoptosis of HUVECs; however, these changes were neutralized by the miR-383-3p inhibitor. The findings indicated that MALAT1 regulates the apoptosis of HUVECs via regulating miR-383-3p expression.

miR-383-5p has been studied in several cancers [47–49]. However, there are no reports on the role of miR-383-5p in DVT. To explore the mechanism by which miR-383-3p affects the apoptosis of HUVECs, we analyzed the potential targets of miR-383-5p, and the data revealed that BCL2L11 is a target of miR-383-5p. BCL2L11 is also called BIM and is a member of the BCL-2 family. It induces apoptosis and inhibits autophagy by inactivating BCL2 or activating BAX-BAK1 and bridging beclin 1 or dynein light chain LC8-type 1 [50–52]. BCL2L11 participates in many biological process [53–55]. Our results indicate that miR-383-5p enhances the viability and inhibits the apoptosis of HUVECs by downregulating BCL2L11 expression levels.

In summary, this study revealed that lncRNA MALAT1 regulated HUVEC apoptosis through regulating the miR-383-5p/BCL2L11 axis in DVT. Nonetheless, it should be noted that this study has some limitations. For example, this study did not explore the correlation between the expression of lncRNA MALAT1 and miR-383-5p in DVT patients at the clinical level. In addition, no animal experiments were performed in this study. We will conduct in-depth exploration and analysis of these issue in the next research.

## Conclusion

We found that the lncRNA MALAT1 regulated HUVEC apoptosis by mediating the miR-383-5p/BCL2L11 axis, thereby playing an important role in DVT. Therefore, the miR-383-5p/BCL2L11 axis may be a potential therapeutic target for the treatment of DVT.

## Supplementary Material

Supplemental Material

## Data Availability

The datasets used and/or analyzed during the present study are available from the corresponding author on reasonable request.
