# Supplementary figures and images for: Significant role of long non-coding RNA MALAT1 in deep vein thrombosis via the regulation of vascular endothelial cell physiology through the microRNA-383-5p/BCL2L11 axis
Source: Bioengineered. 2022 Jun 15;13(5):13728–38. doi: 10.1080/21655979.2022.2080412 (PMC9276002; doi:10.1080/21655979.2022.2080412)

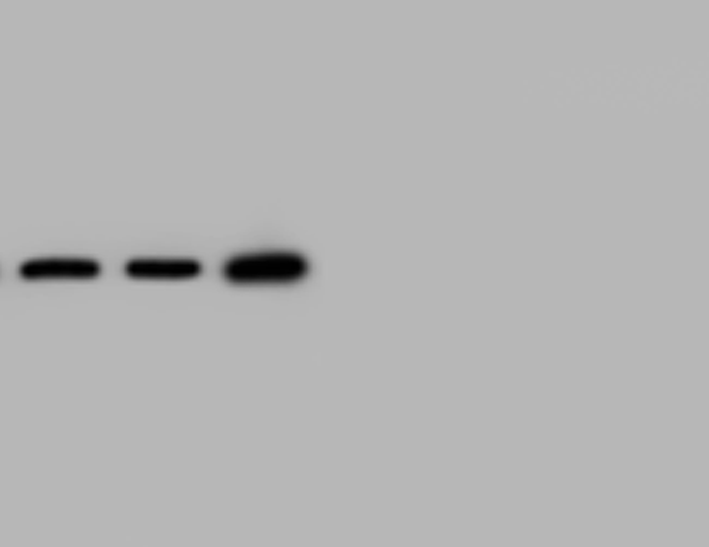

Supplement: Supplemental Material [file KBIE_A_2080412_SM3012.zip › Supplementary materials/Original blots-Figure 1/Bax.tif]

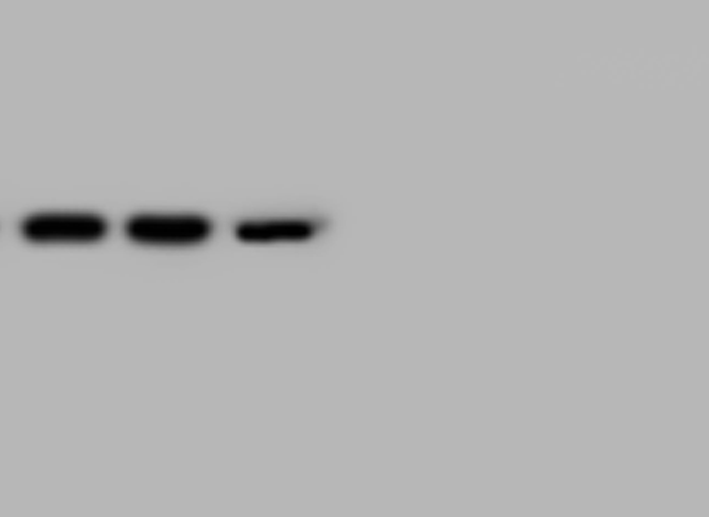

Supplement: Supplemental Material [file KBIE_A_2080412_SM3012.zip › Supplementary materials/Original blots-Figure 1/Bcl-2.tif]

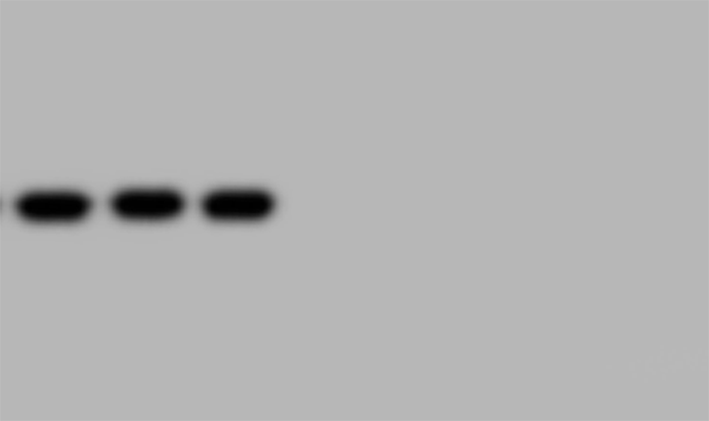

Supplement: Supplemental Material [file KBIE_A_2080412_SM3012.zip › Supplementary materials/Original blots-Figure 1/GAPDH.tif]

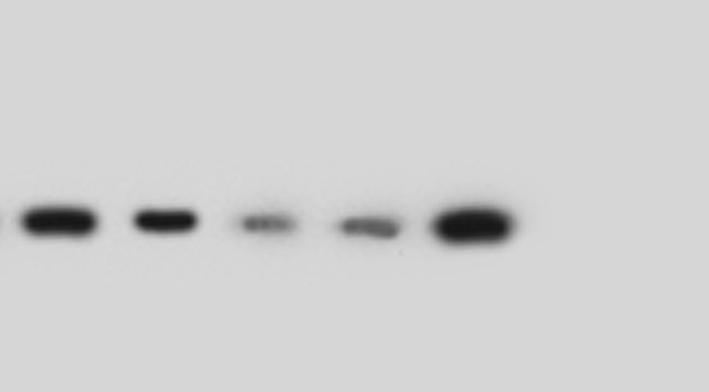

Supplement: Supplemental Material [file KBIE_A_2080412_SM3012.zip › Supplementary materials/Original blots-Figure 4/Bax.tif]

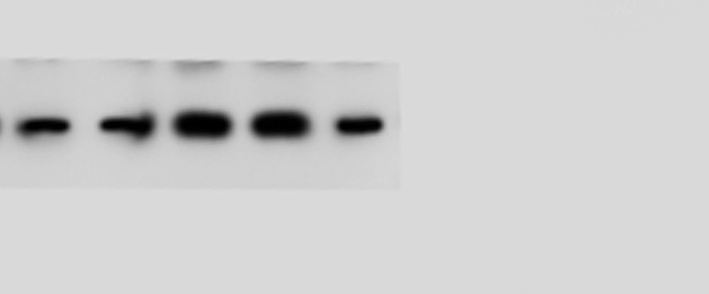

Supplement: Supplemental Material [file KBIE_A_2080412_SM3012.zip › Supplementary materials/Original blots-Figure 4/Bcl-2.tif]

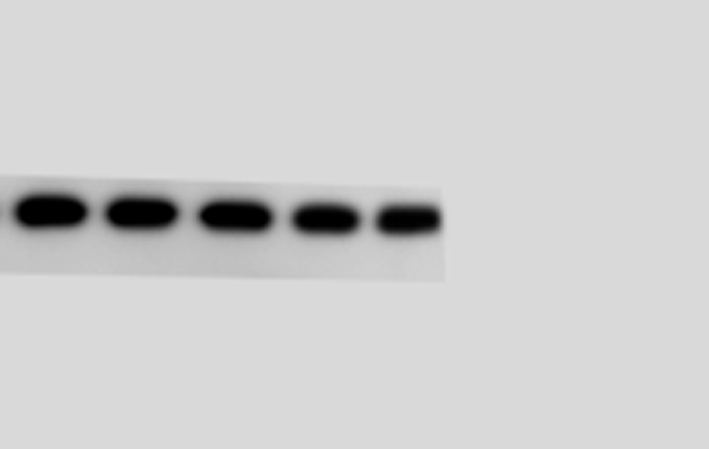

Supplement: Supplemental Material [file KBIE_A_2080412_SM3012.zip › Supplementary materials/Original blots-Figure 4/GAPDH.tif]

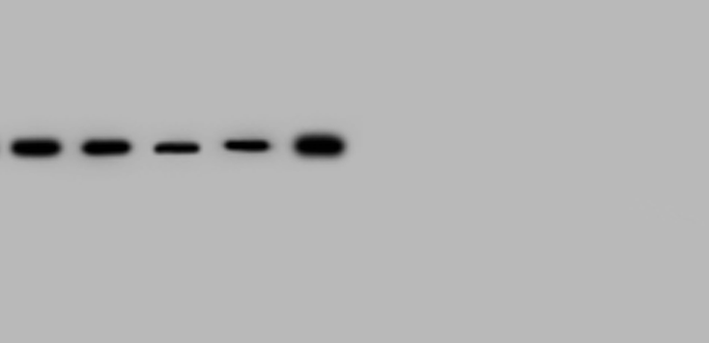

Supplement: Supplemental Material [file KBIE_A_2080412_SM3012.zip › Supplementary materials/Original blots-Figure 6/BCL2L11.tif]

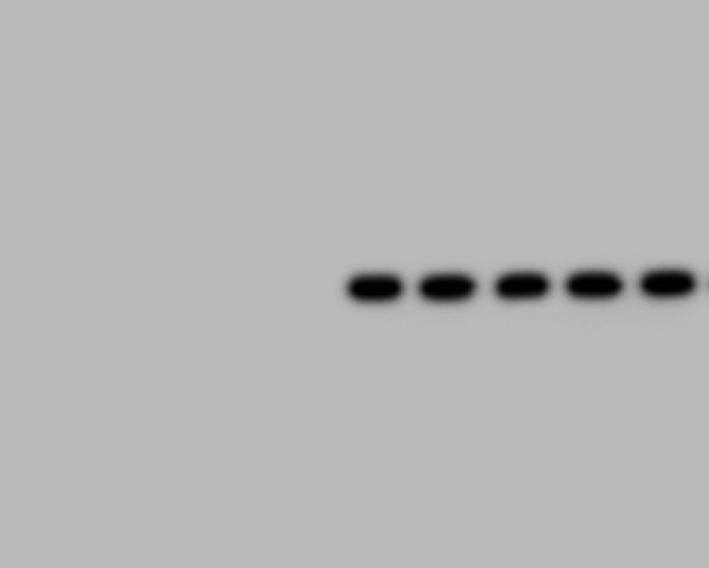

Supplement: Supplemental Material [file KBIE_A_2080412_SM3012.zip › Supplementary materials/Original blots-Figure 6/GAPDH.tif]

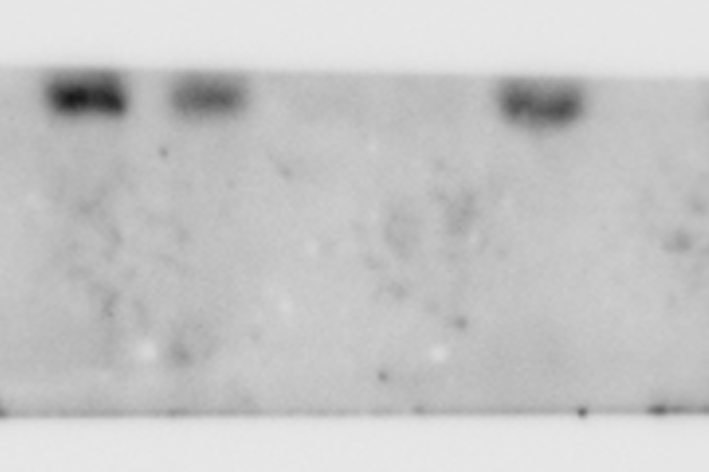

Supplement: Supplemental Material [file KBIE_A_2080412_SM3012.zip › Supplementary materials/Original blots-Figure 7/Bax.tif]

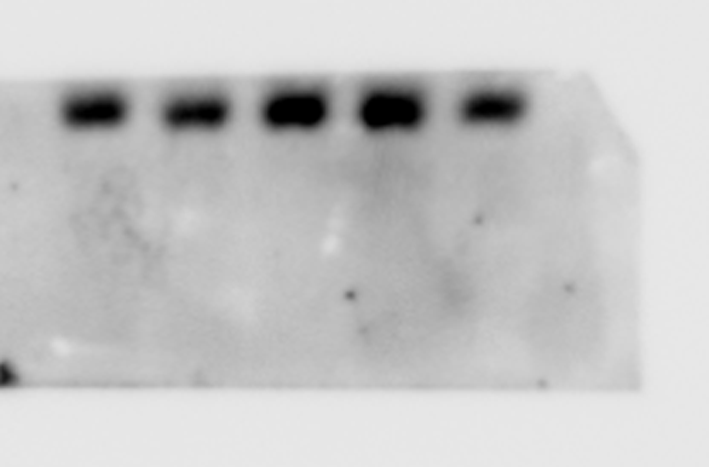

Supplement: Supplemental Material [file KBIE_A_2080412_SM3012.zip › Supplementary materials/Original blots-Figure 7/Bcl-2.tif]

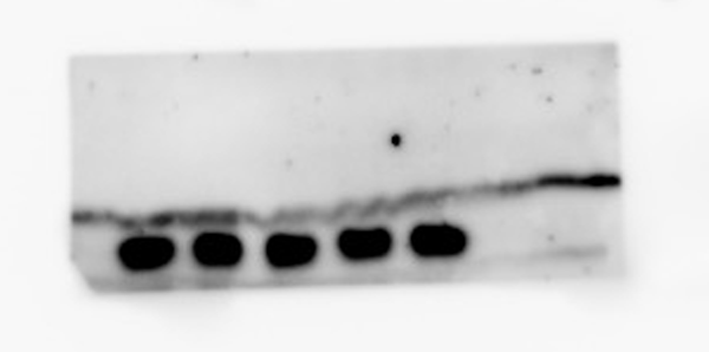

Supplement: Supplemental Material [file KBIE_A_2080412_SM3012.zip › Supplementary materials/Original blots-Figure 7/GAPDH.tif]

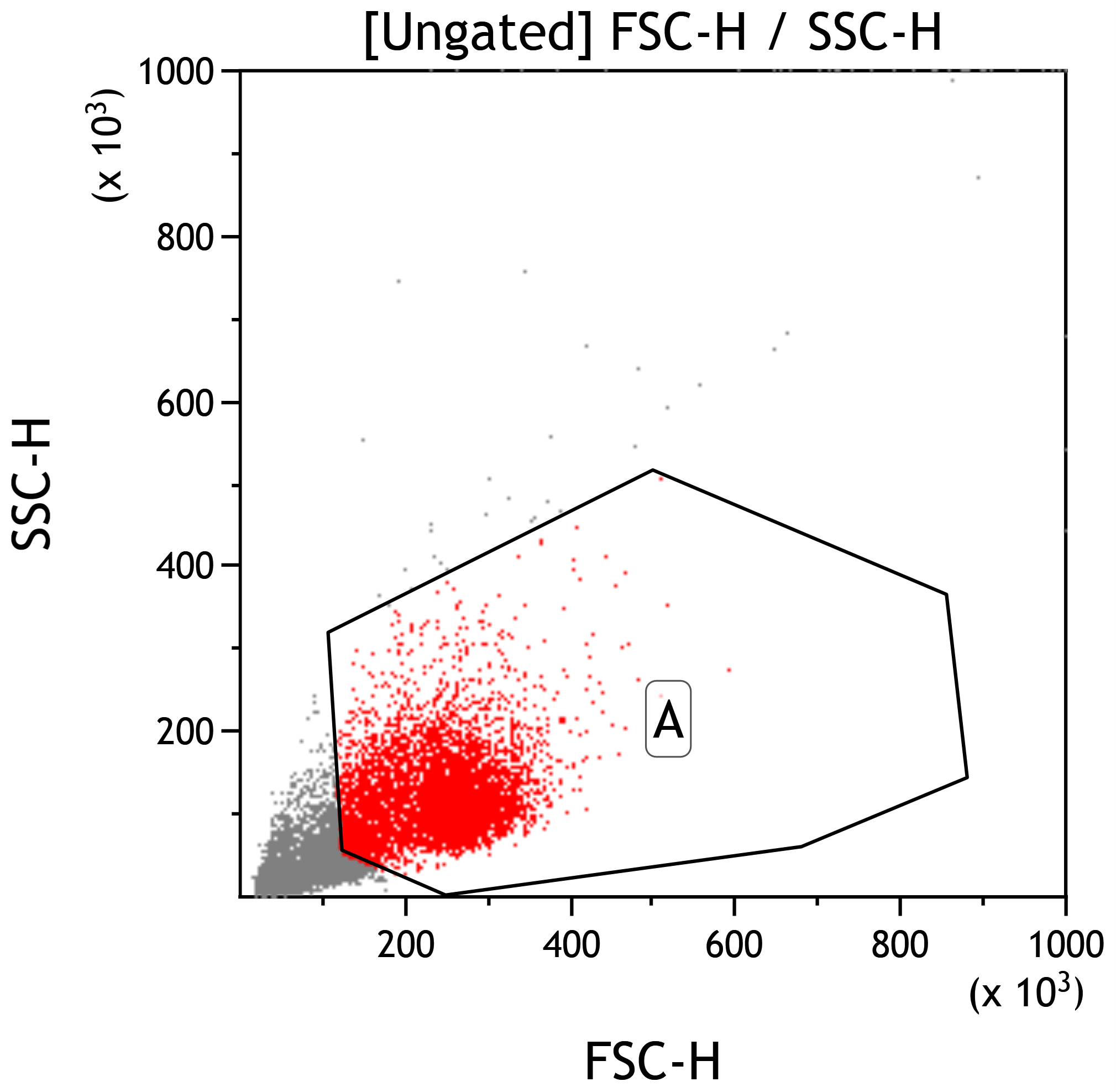

Supplement: Supplemental Material [file KBIE_A_2080412_SM3012.zip › Supplementary materials/apoptosis-FCM/FCM-Figure 1/Control-1.png]

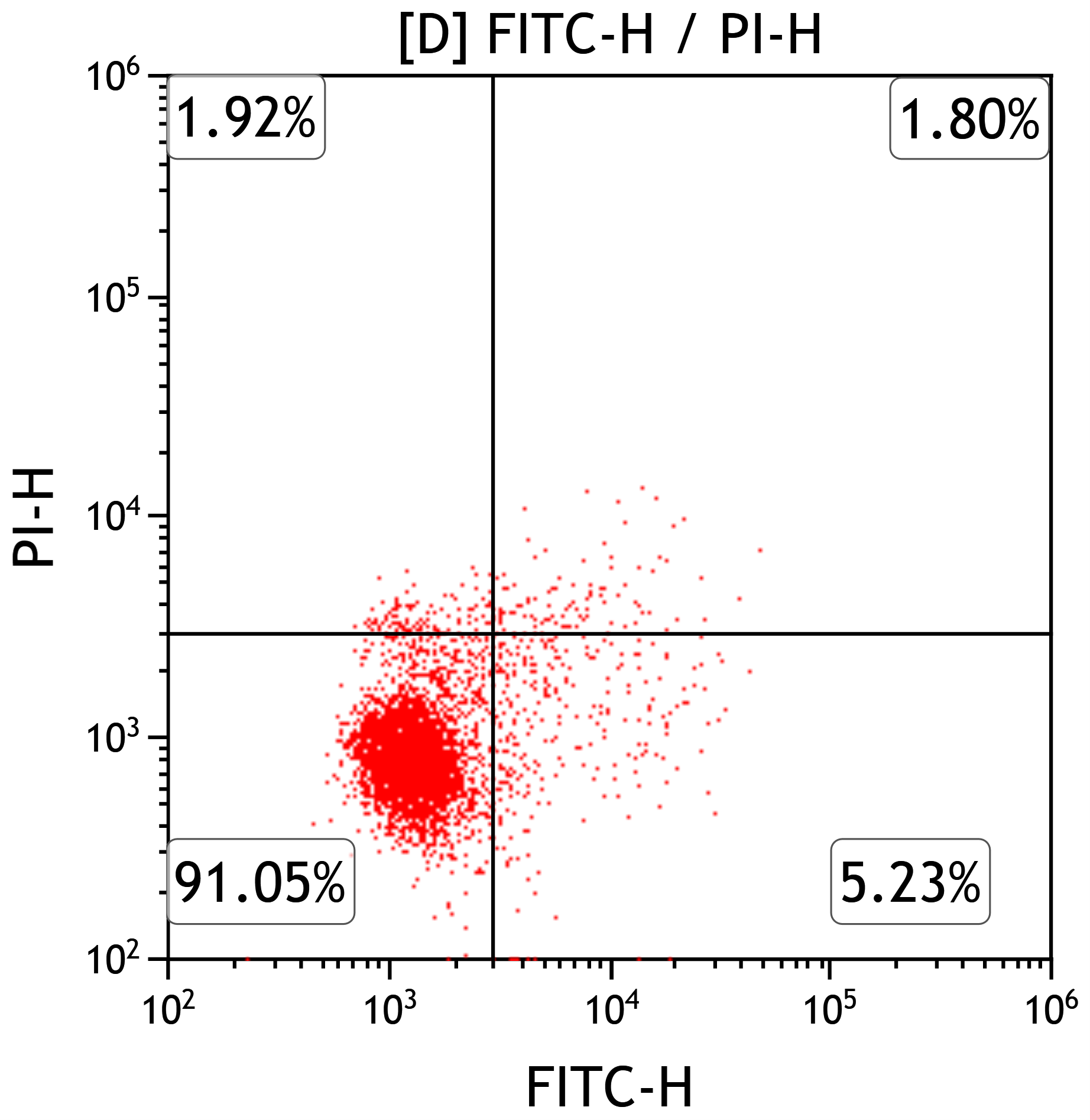

Supplement: Supplemental Material [file KBIE_A_2080412_SM3012.zip › Supplementary materials/apoptosis-FCM/FCM-Figure 1/Control-2.png]

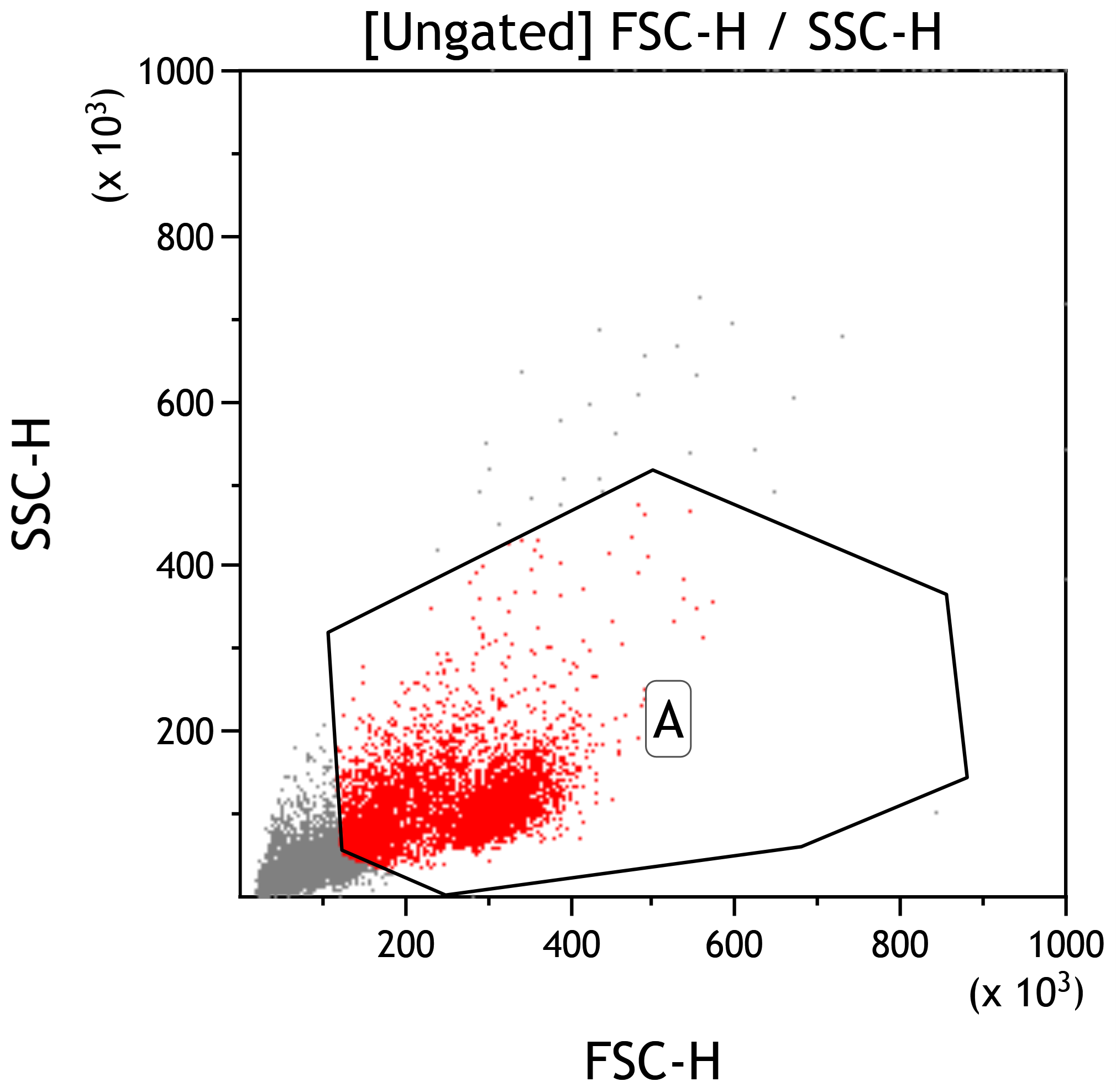

Supplement: Supplemental Material [file KBIE_A_2080412_SM3012.zip › Supplementary materials/apoptosis-FCM/FCM-Figure 1/Control-plasmid-1.png]

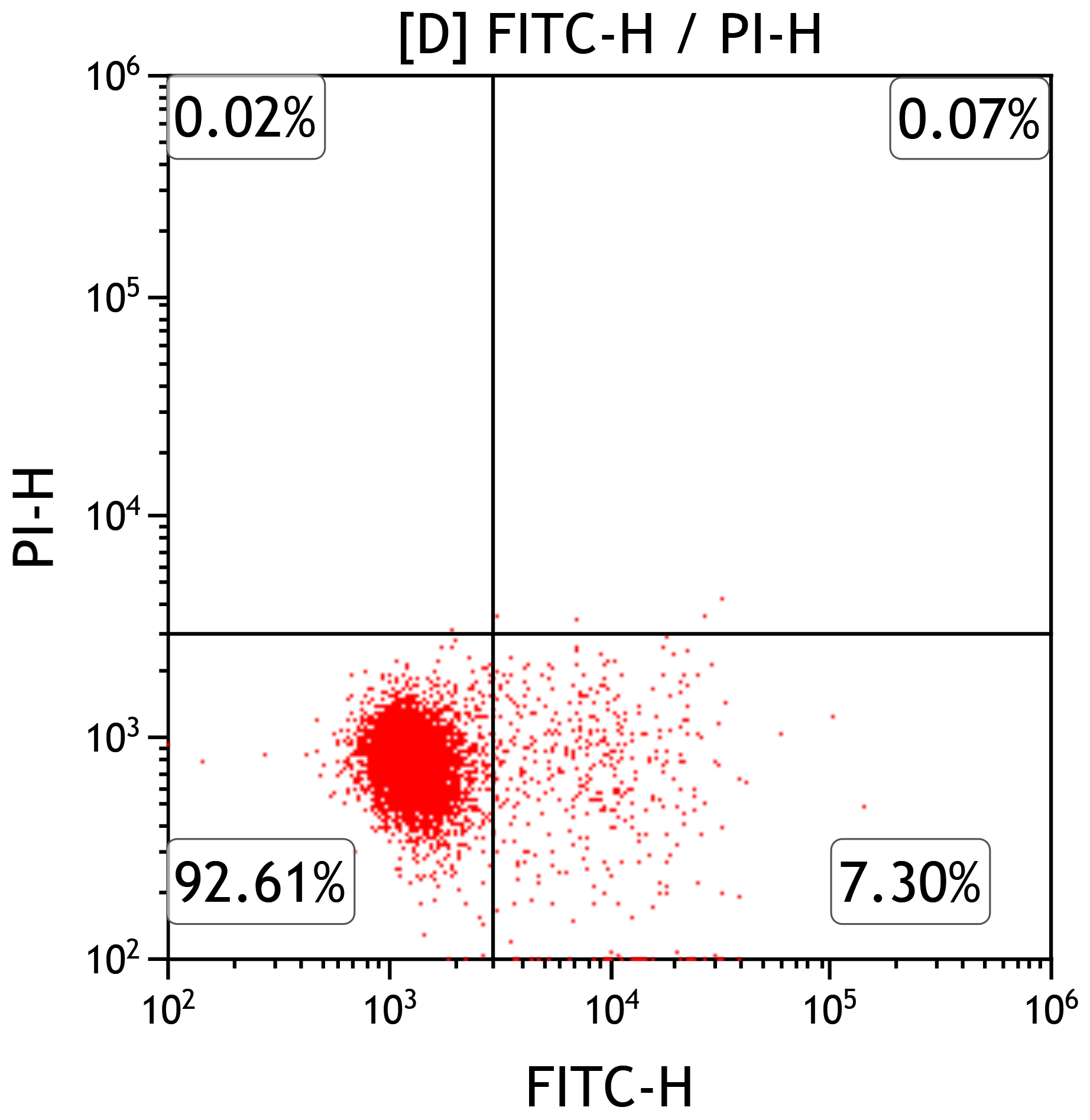

Supplement: Supplemental Material [file KBIE_A_2080412_SM3012.zip › Supplementary materials/apoptosis-FCM/FCM-Figure 1/Control-plasmid-2.png]

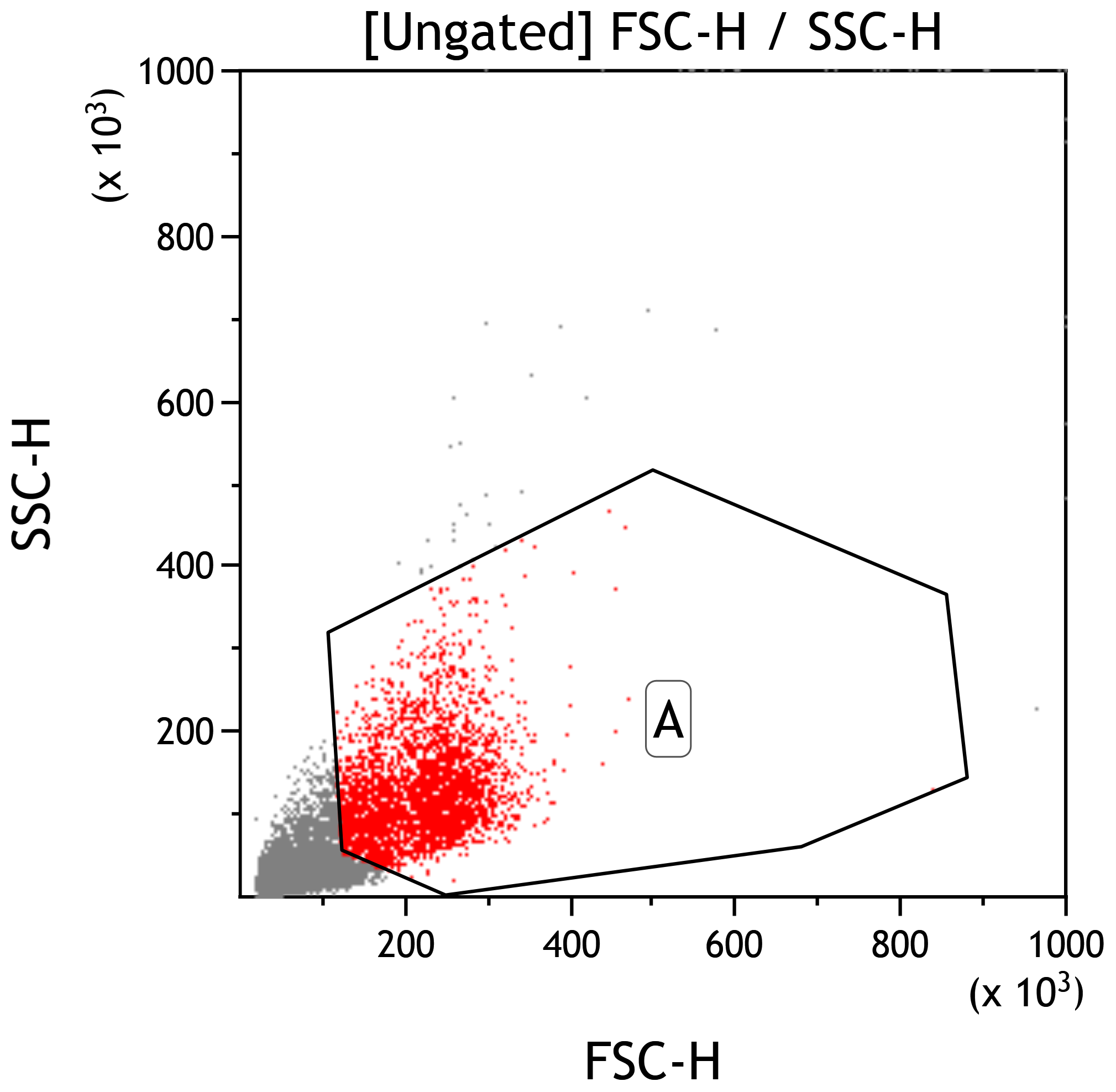

Supplement: Supplemental Material [file KBIE_A_2080412_SM3012.zip › Supplementary materials/apoptosis-FCM/FCM-Figure 1/MALAT1-plasmid-1.png]

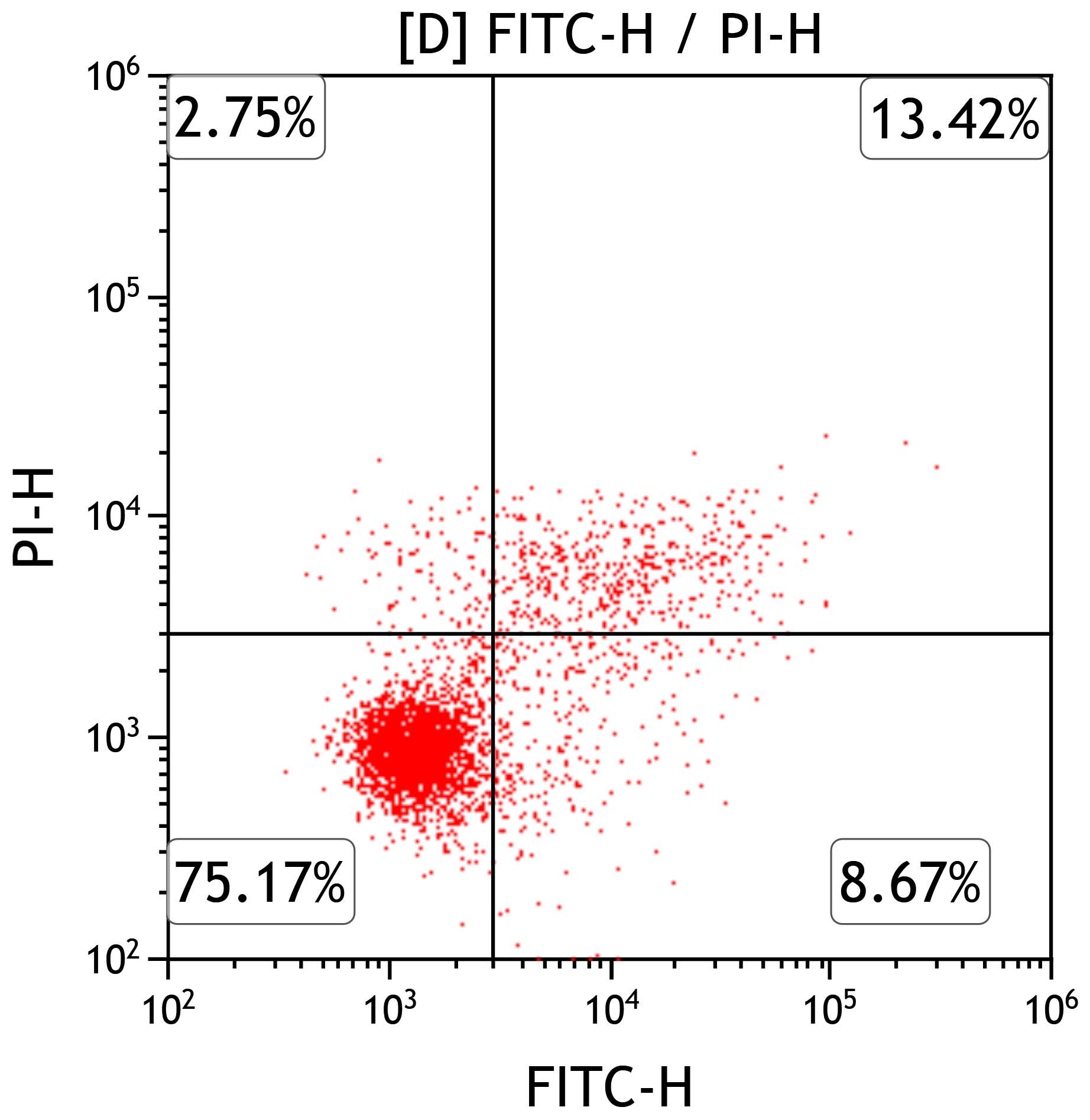

Supplement: Supplemental Material [file KBIE_A_2080412_SM3012.zip › Supplementary materials/apoptosis-FCM/FCM-Figure 1/MALAT1-plasmid-2.png]

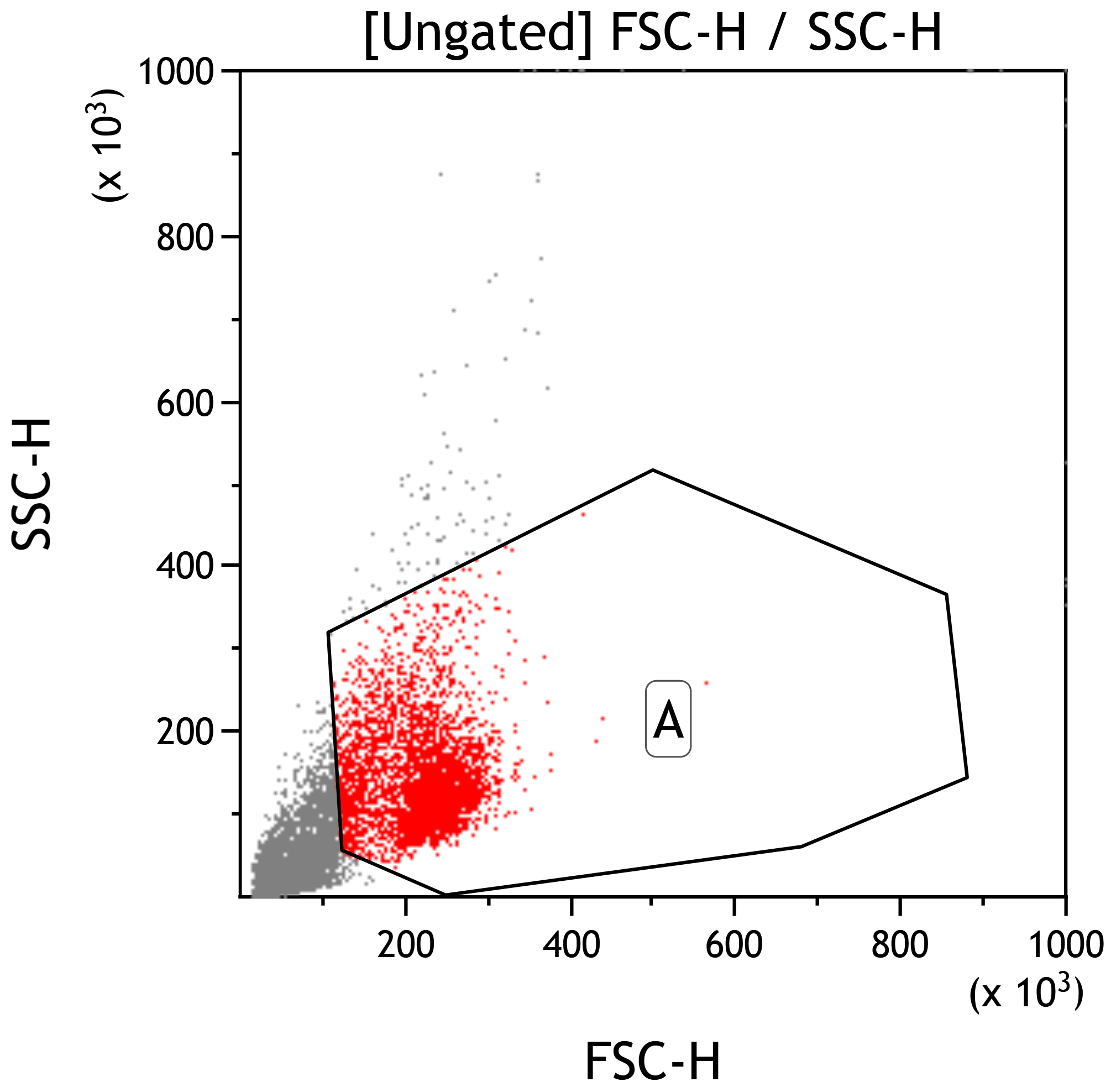

Supplement: Supplemental Material [file KBIE_A_2080412_SM3012.zip › Supplementary materials/apoptosis-FCM/FCM-Figure 4/Control-1.png]

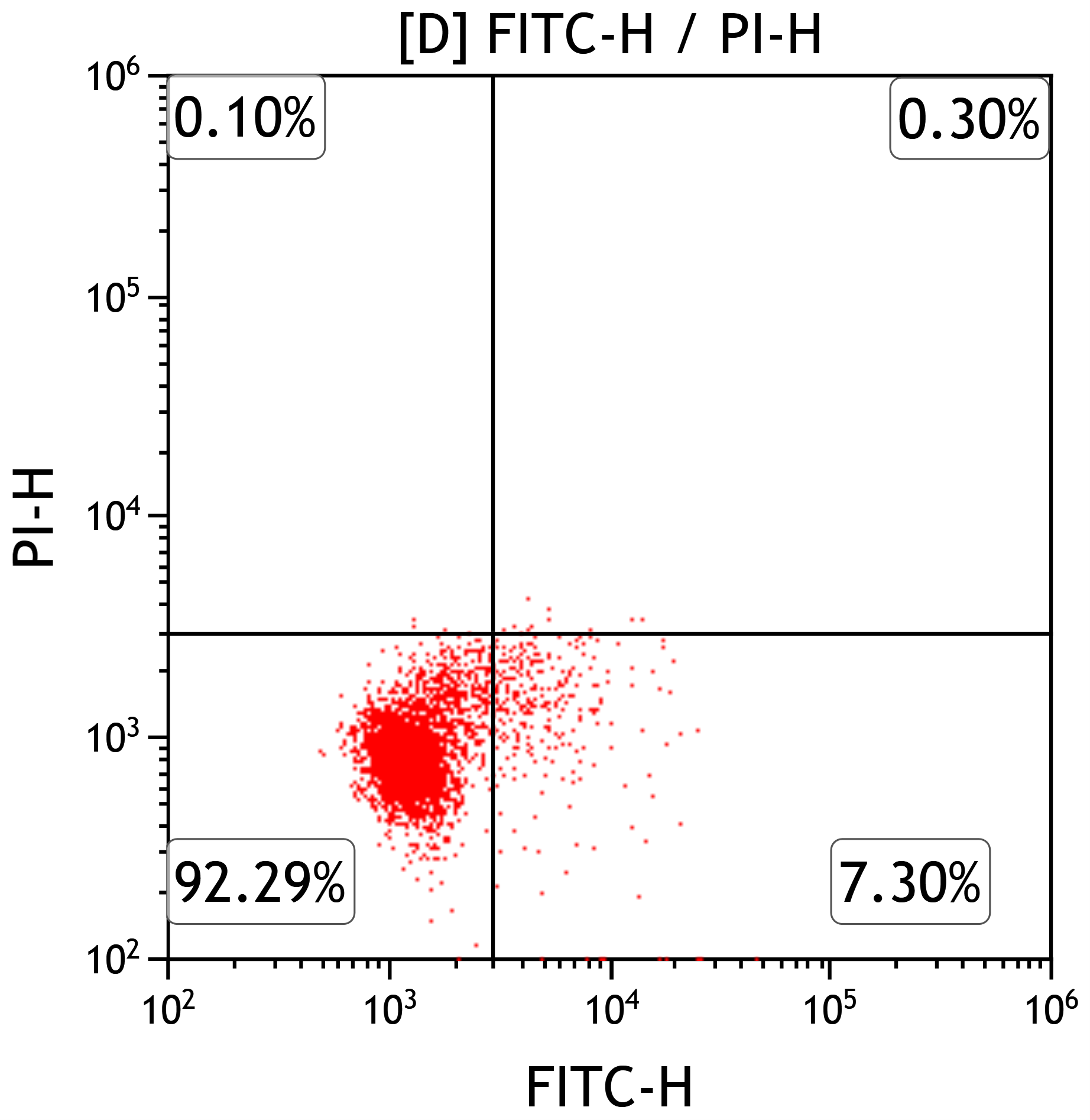

Supplement: Supplemental Material [file KBIE_A_2080412_SM3012.zip › Supplementary materials/apoptosis-FCM/FCM-Figure 4/Control-2.png]

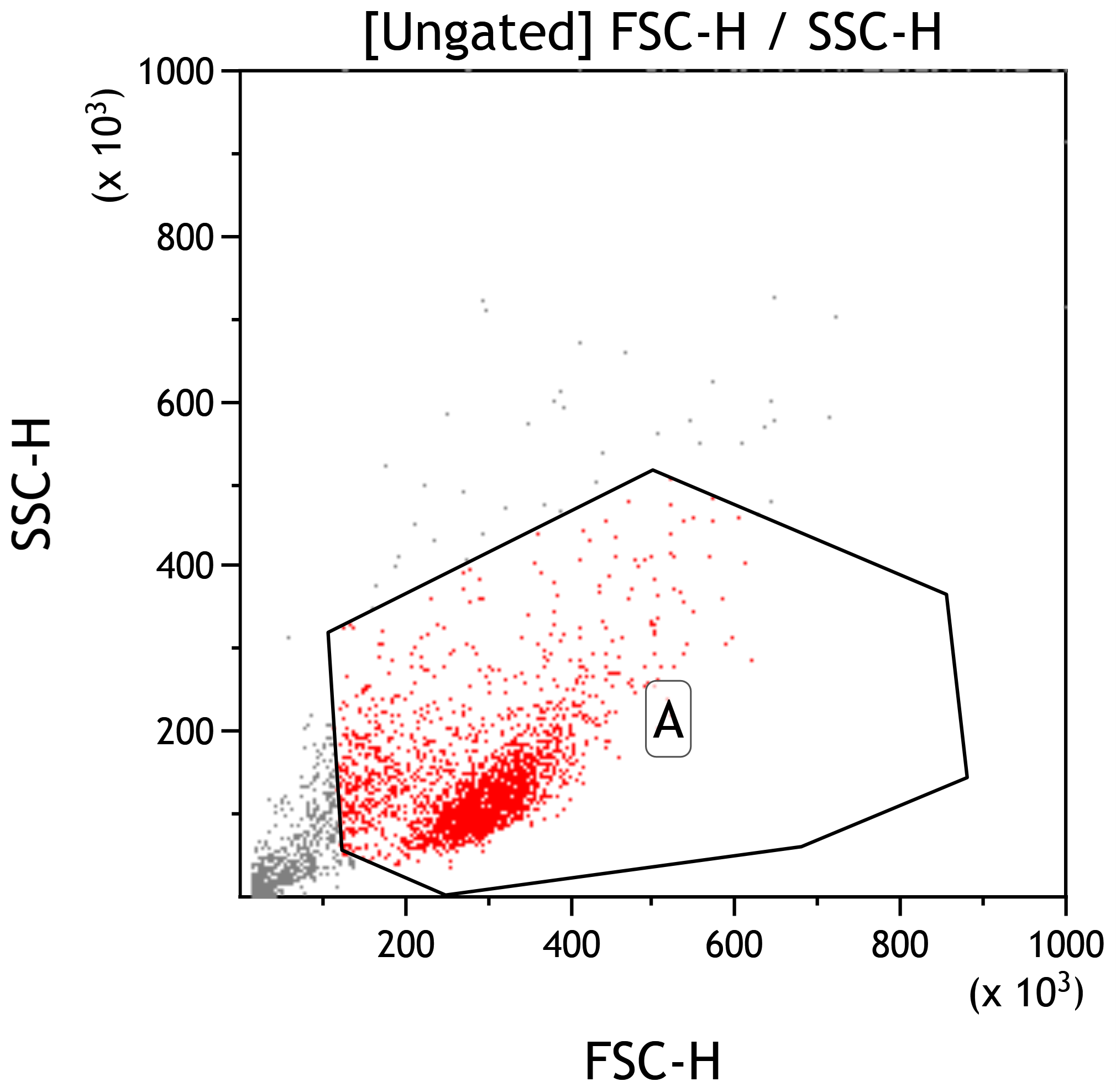

Supplement: Supplemental Material [file KBIE_A_2080412_SM3012.zip › Supplementary materials/apoptosis-FCM/FCM-Figure 4/MALAT1-siRNA+inhibitor control-1.png]

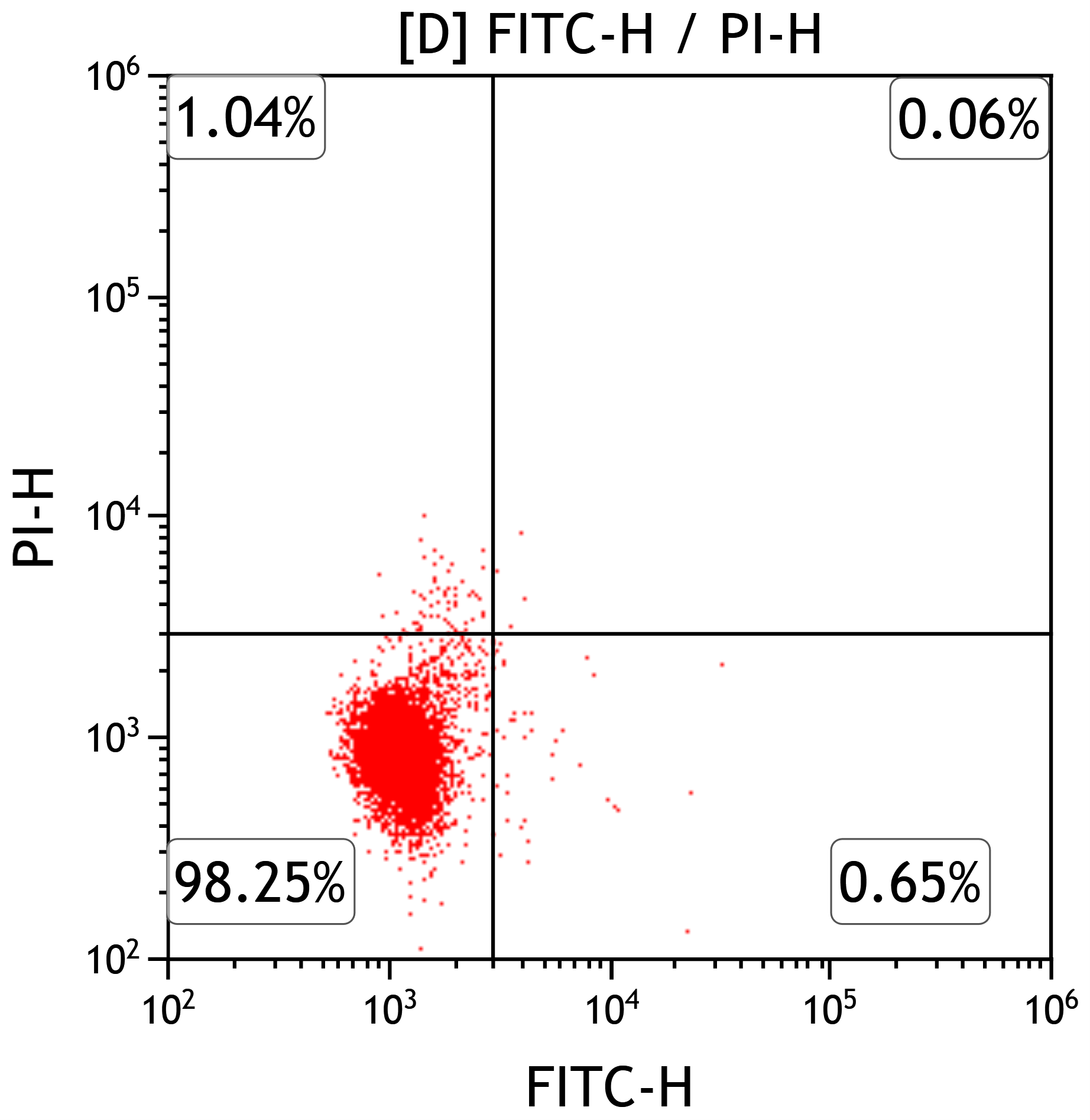

Supplement: Supplemental Material [file KBIE_A_2080412_SM3012.zip › Supplementary materials/apoptosis-FCM/FCM-Figure 4/MALAT1-siRNA+inhibitor control-2.png]

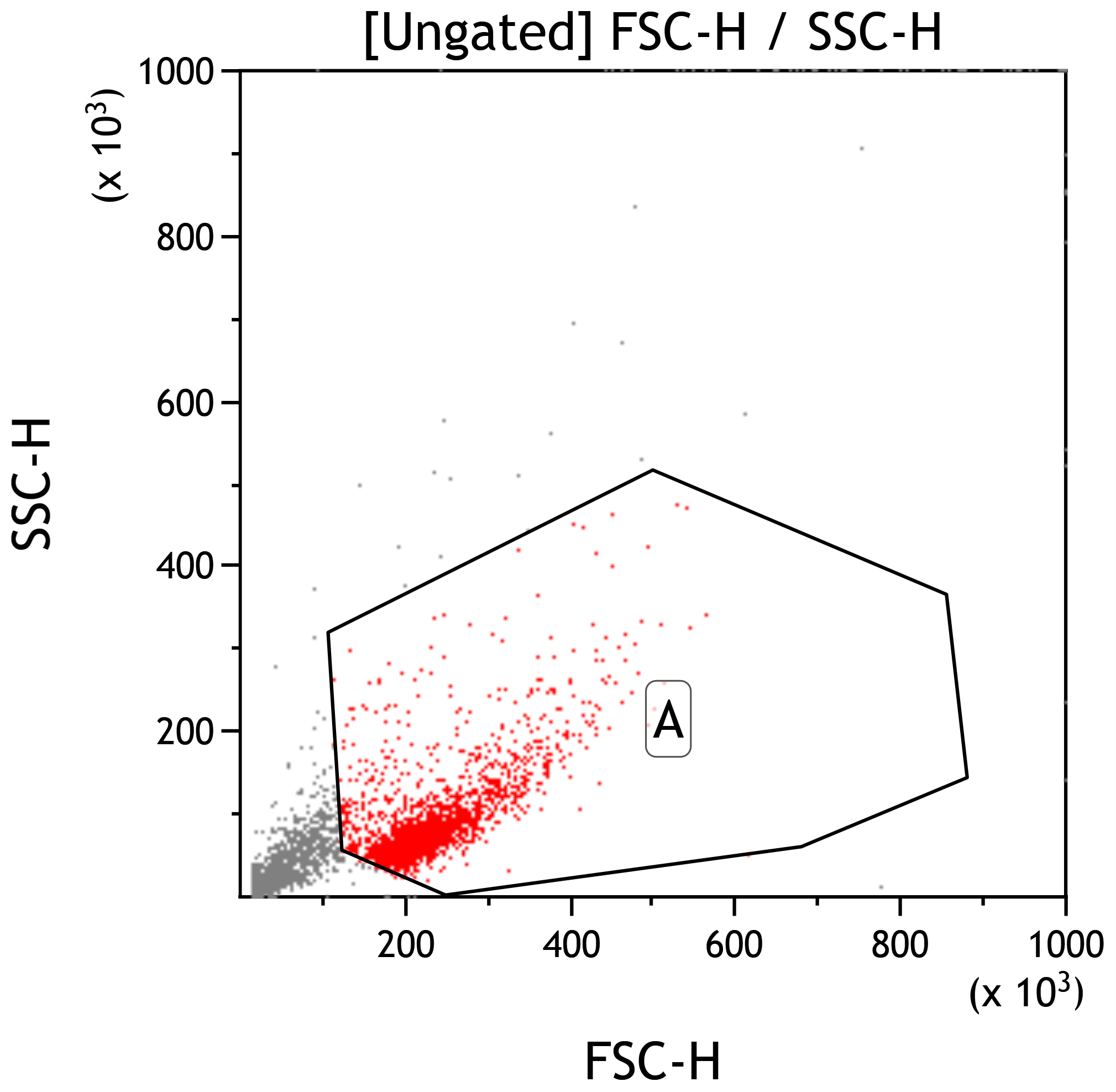

Supplement: Supplemental Material [file KBIE_A_2080412_SM3012.zip › Supplementary materials/apoptosis-FCM/FCM-Figure 4/MALAT1-siRNA+miR-383-5p inhibitor-1.png]

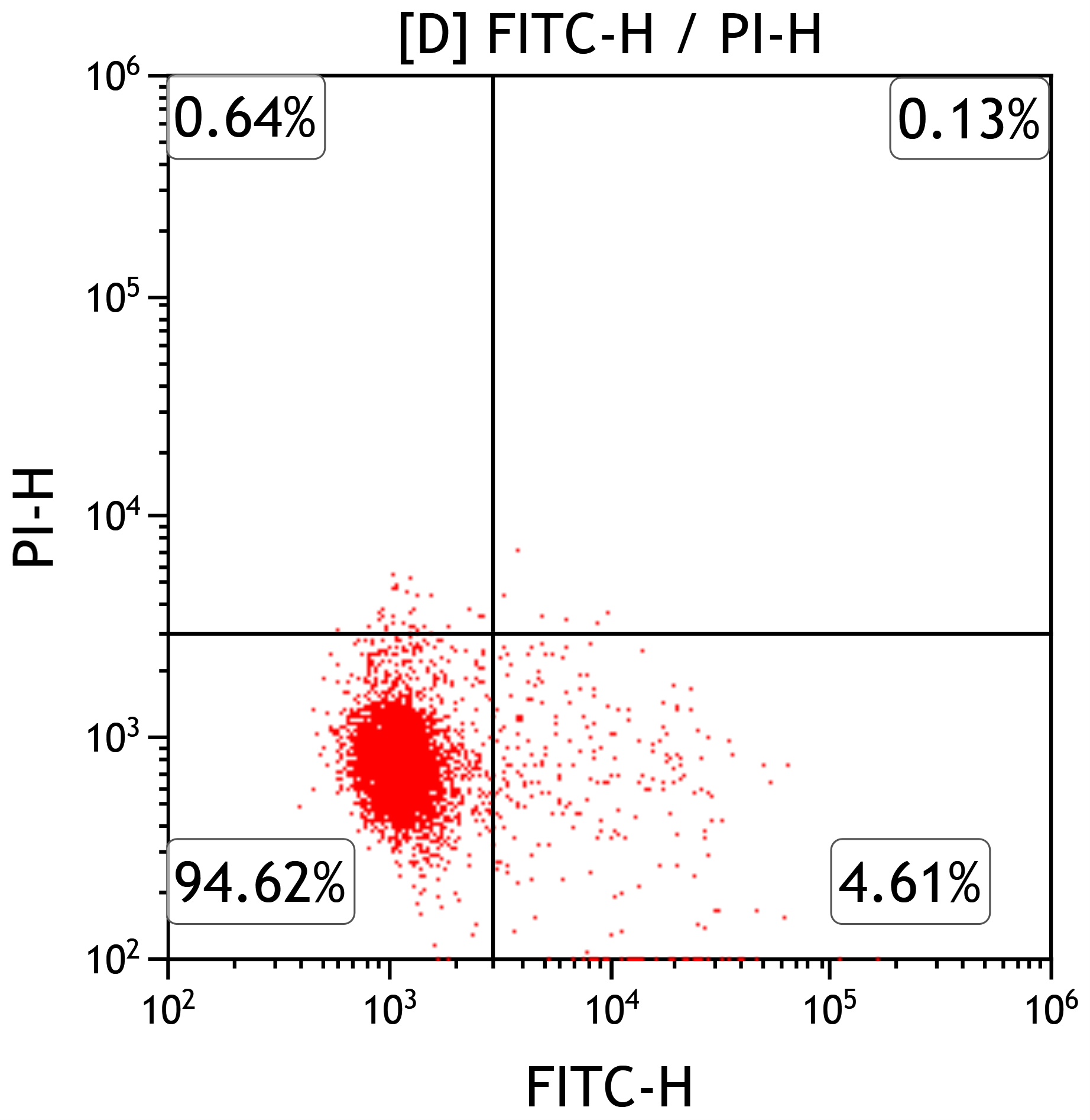

Supplement: Supplemental Material [file KBIE_A_2080412_SM3012.zip › Supplementary materials/apoptosis-FCM/FCM-Figure 4/MALAT1-siRNA+miR-383-5p inhibitor-2.png]

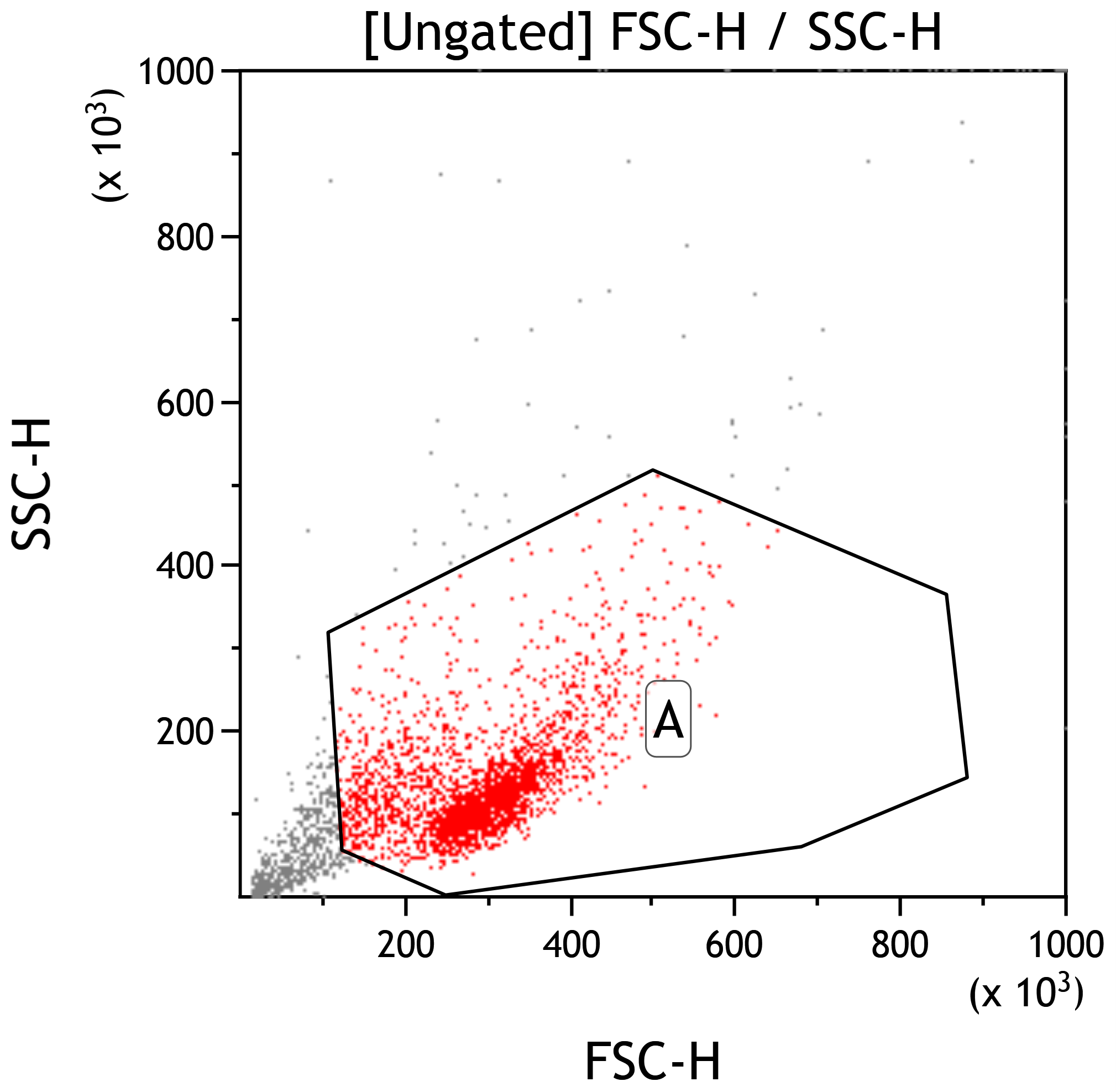

Supplement: Supplemental Material [file KBIE_A_2080412_SM3012.zip › Supplementary materials/apoptosis-FCM/FCM-Figure 4/MALAT1-siRNA-1.png]

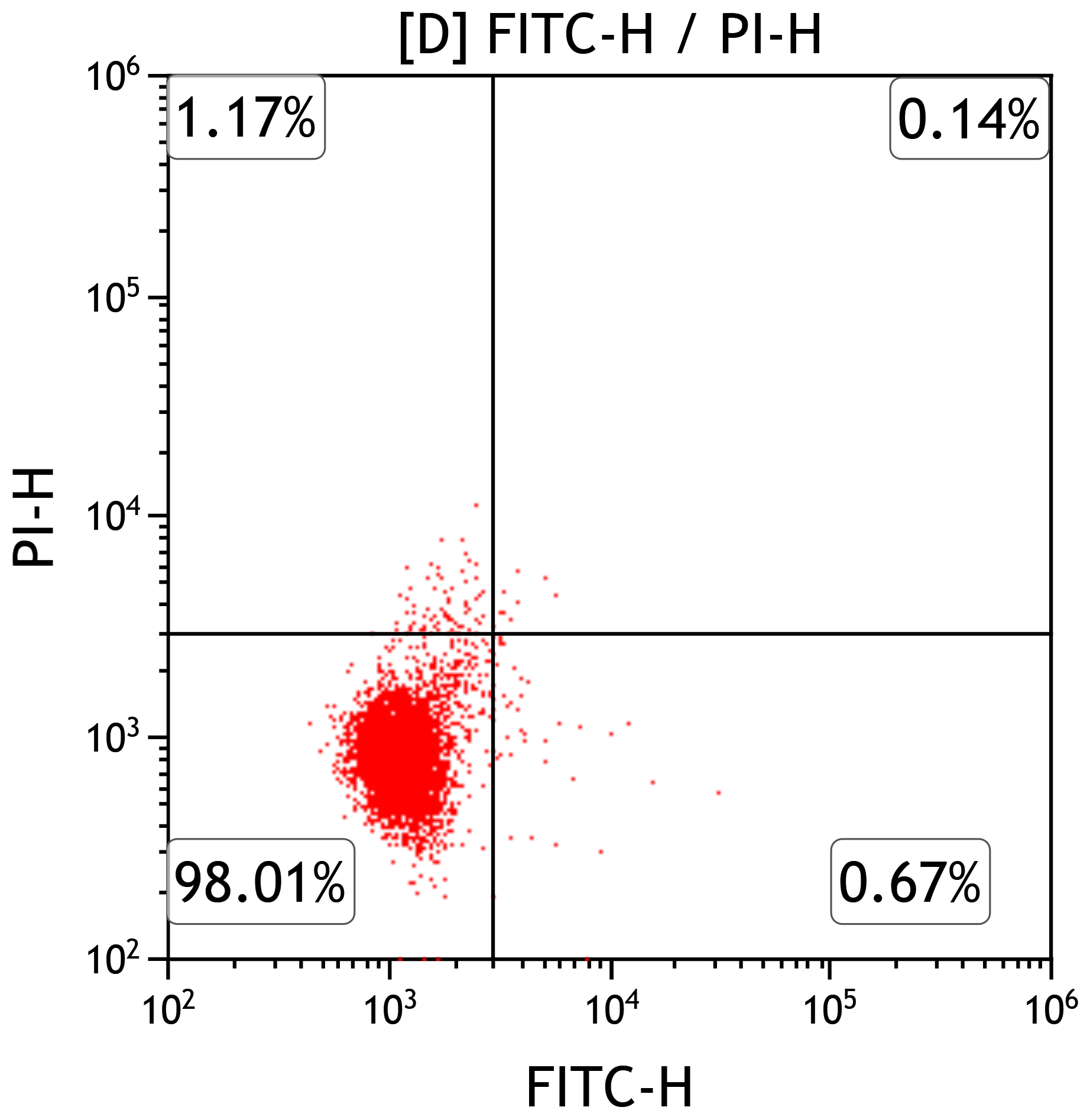

Supplement: Supplemental Material [file KBIE_A_2080412_SM3012.zip › Supplementary materials/apoptosis-FCM/FCM-Figure 4/MALAT1-siRNA-2.png]

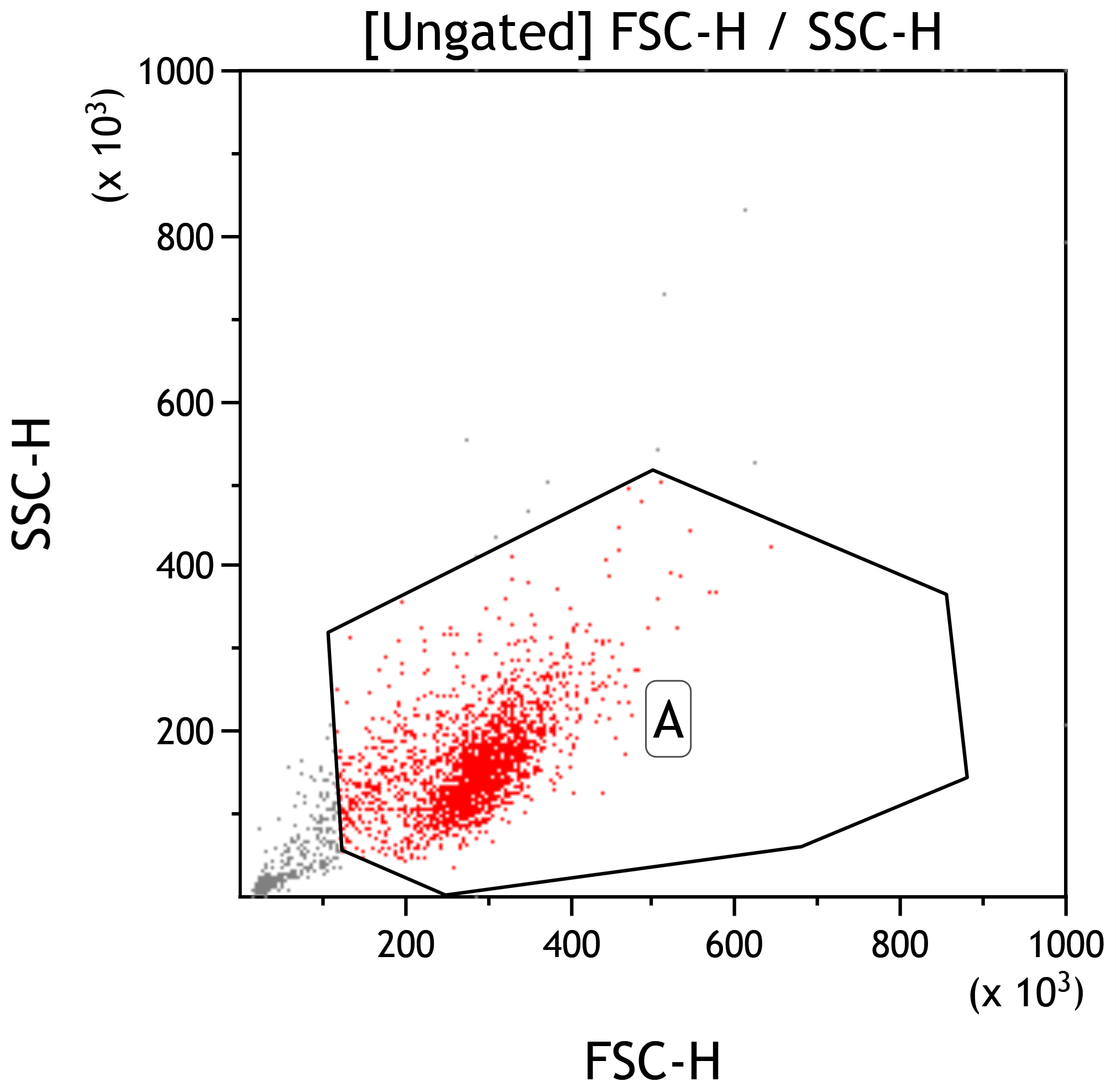

Supplement: Supplemental Material [file KBIE_A_2080412_SM3012.zip › Supplementary materials/apoptosis-FCM/FCM-Figure 4/control-siRNA-1.png]

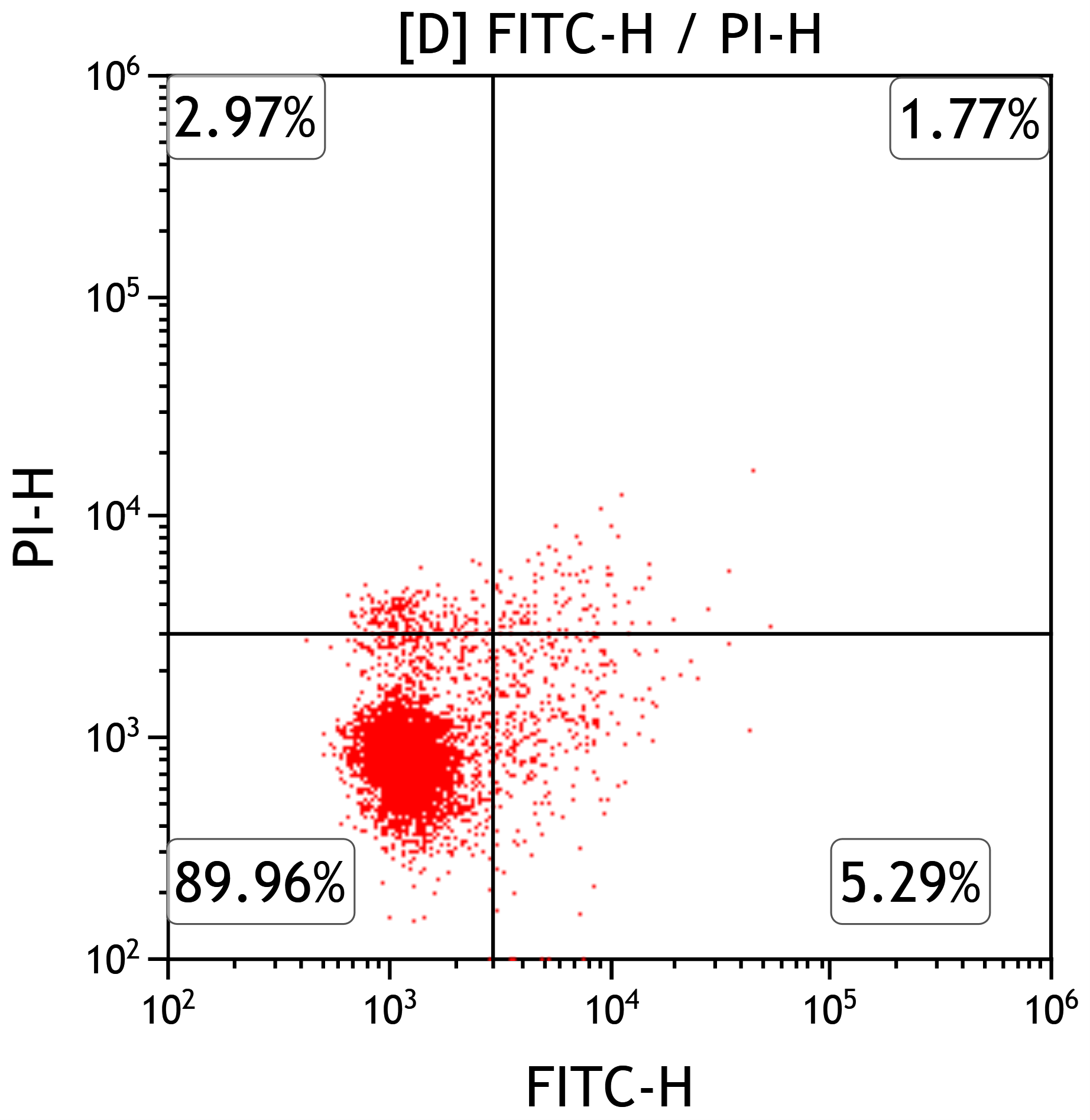

Supplement: Supplemental Material [file KBIE_A_2080412_SM3012.zip › Supplementary materials/apoptosis-FCM/FCM-Figure 4/control-siRNA-2.png]

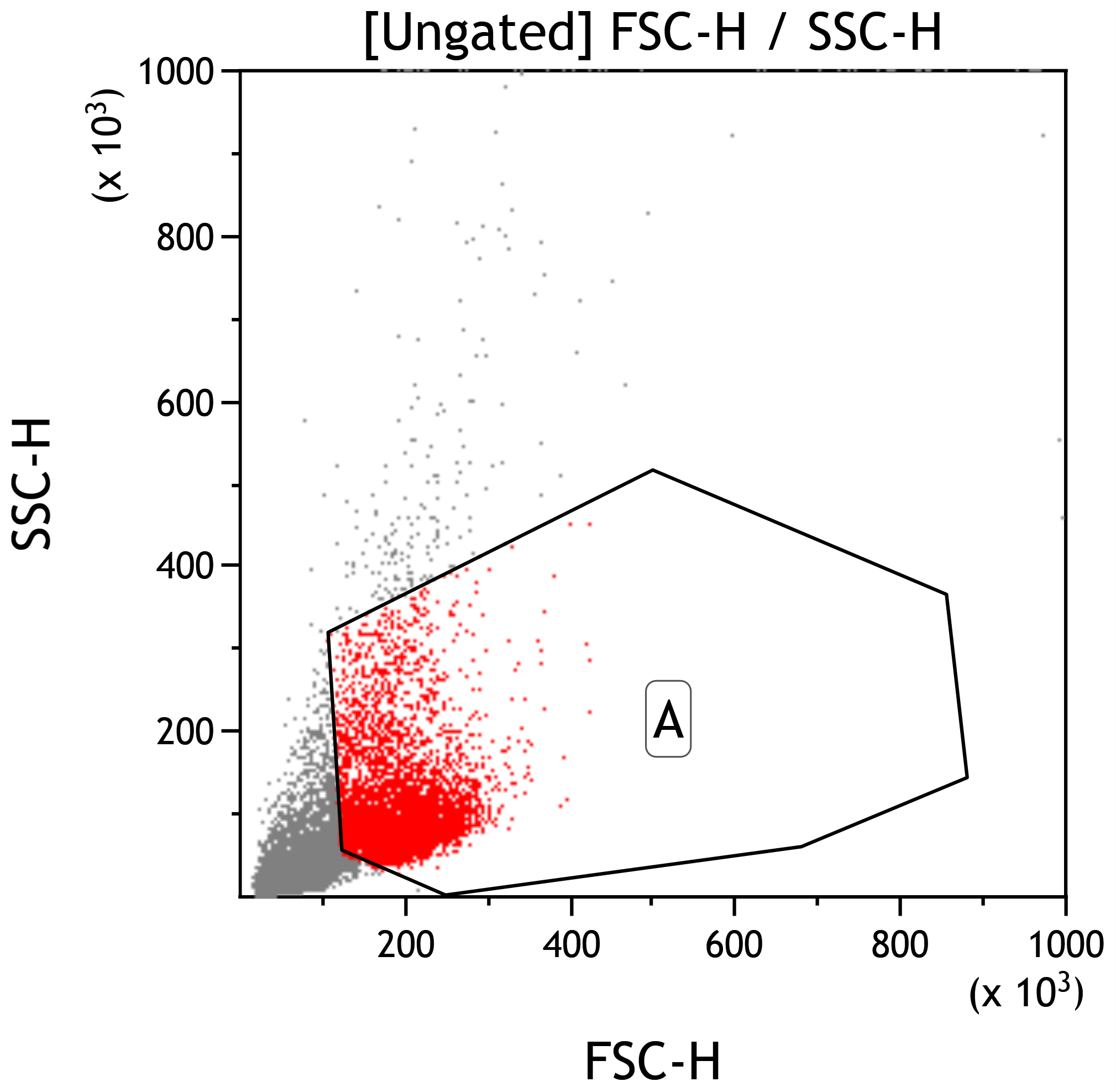

Supplement: Supplemental Material [file KBIE_A_2080412_SM3012.zip › Supplementary materials/apoptosis-FCM/FCM-Figure 7/Control-1.png]

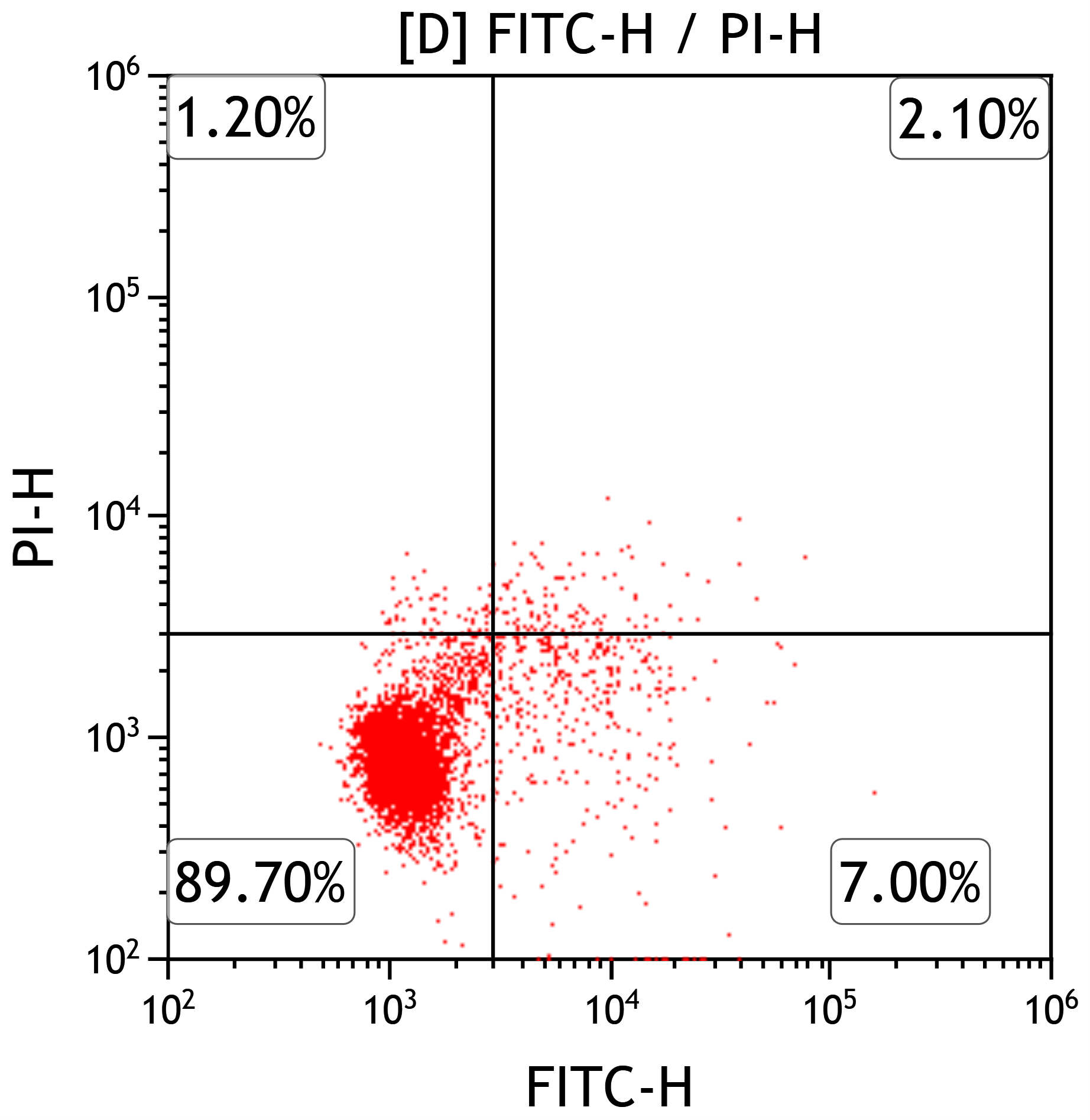

Supplement: Supplemental Material [file KBIE_A_2080412_SM3012.zip › Supplementary materials/apoptosis-FCM/FCM-Figure 7/Control-2.png]

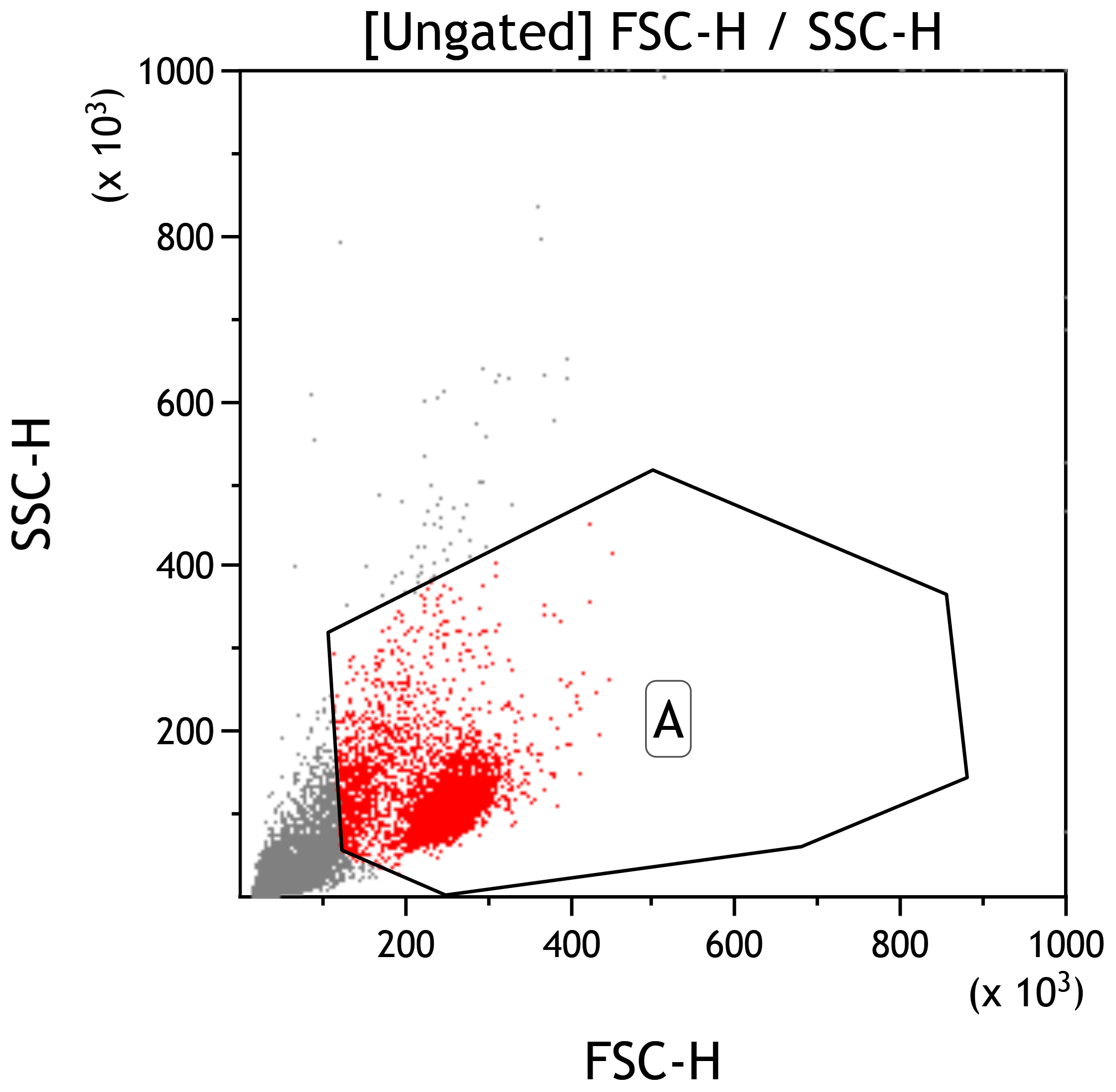

Supplement: Supplemental Material [file KBIE_A_2080412_SM3012.zip › Supplementary materials/apoptosis-FCM/FCM-Figure 7/miR-383-5p mimic+BCL2L11-plasmid-1.png]

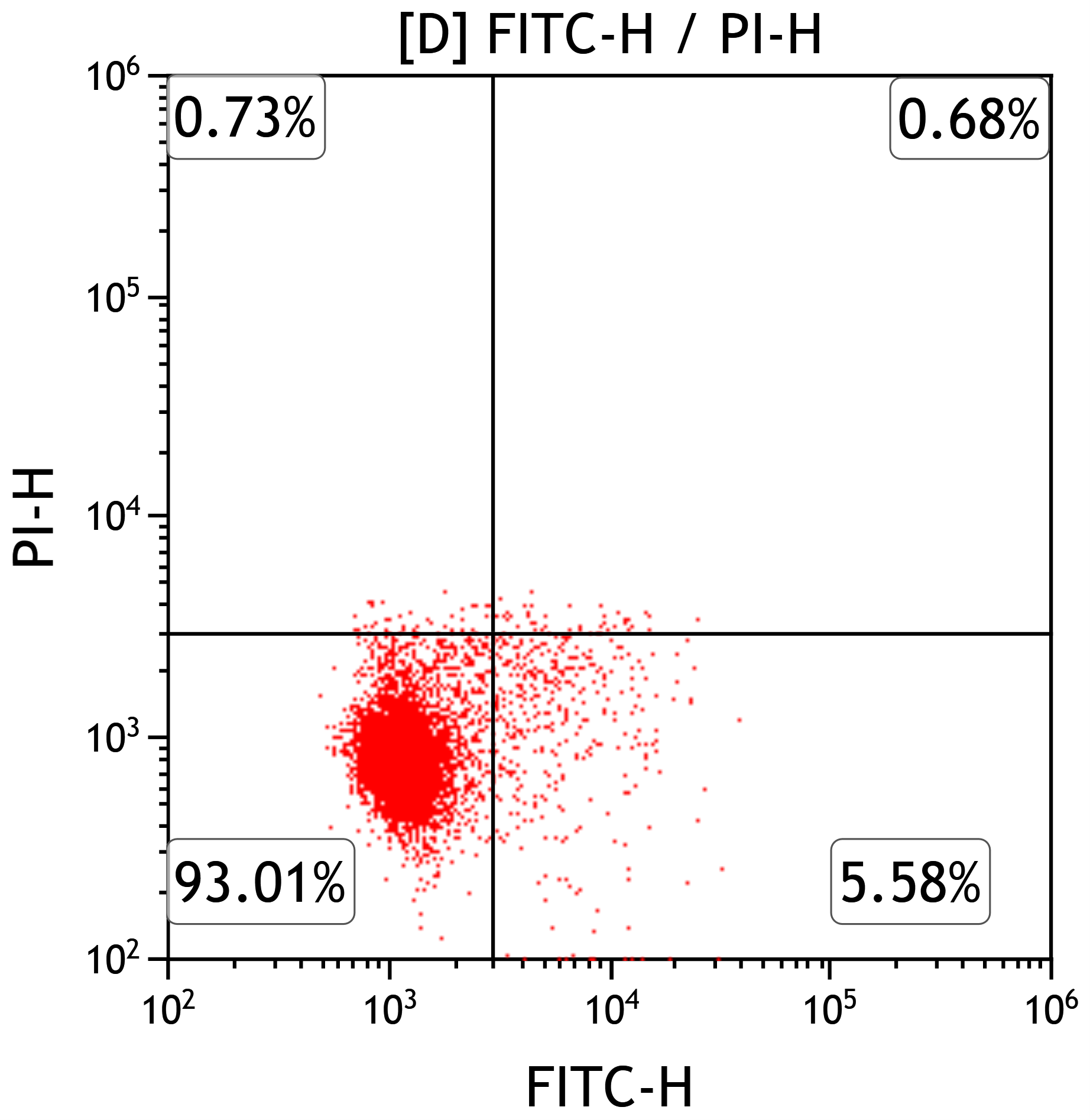

Supplement: Supplemental Material [file KBIE_A_2080412_SM3012.zip › Supplementary materials/apoptosis-FCM/FCM-Figure 7/miR-383-5p mimic+BCL2L11-plasmid-2.png]

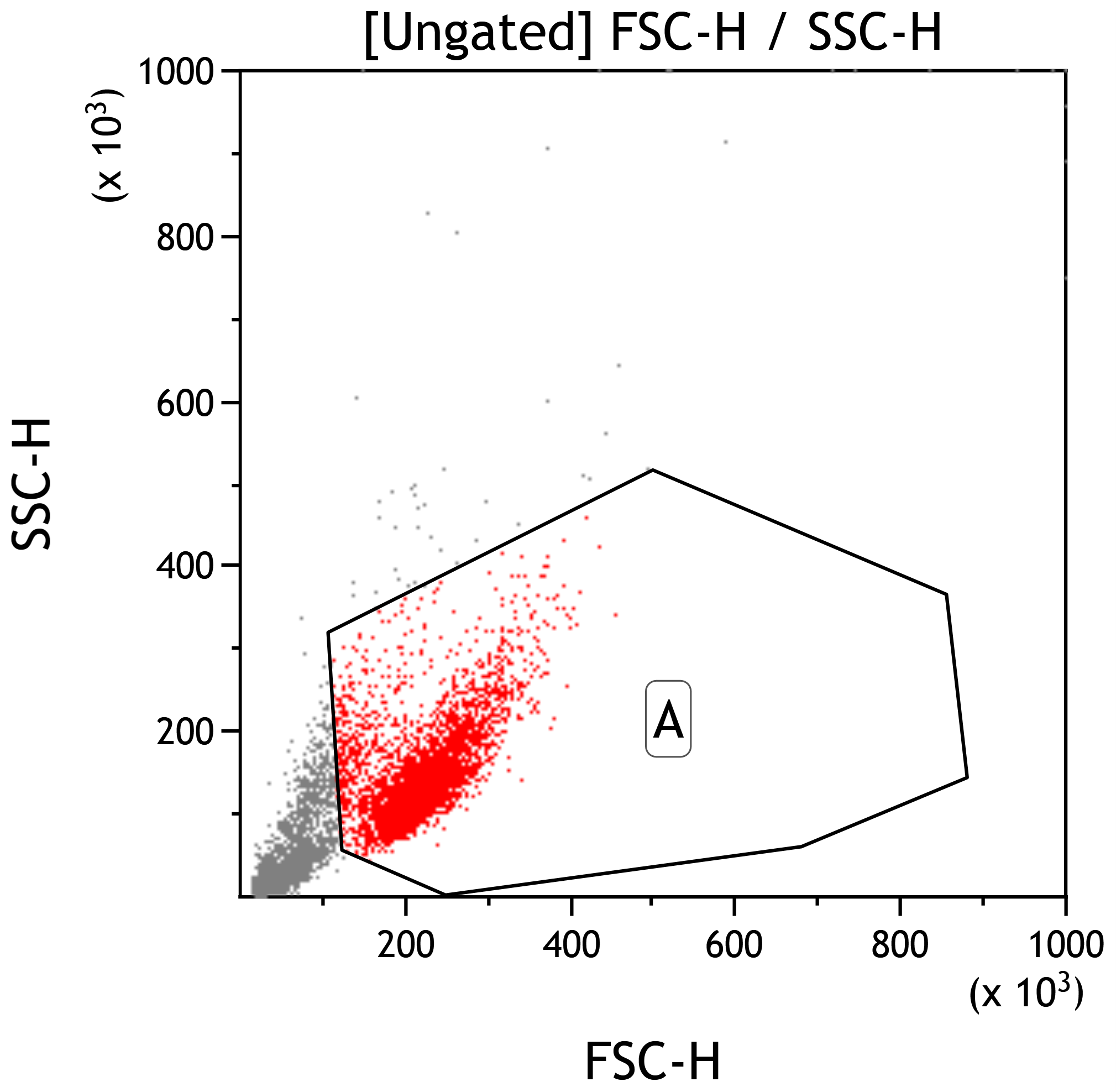

Supplement: Supplemental Material [file KBIE_A_2080412_SM3012.zip › Supplementary materials/apoptosis-FCM/FCM-Figure 7/miR-383-5p mimic+control-plasmid-1.png]

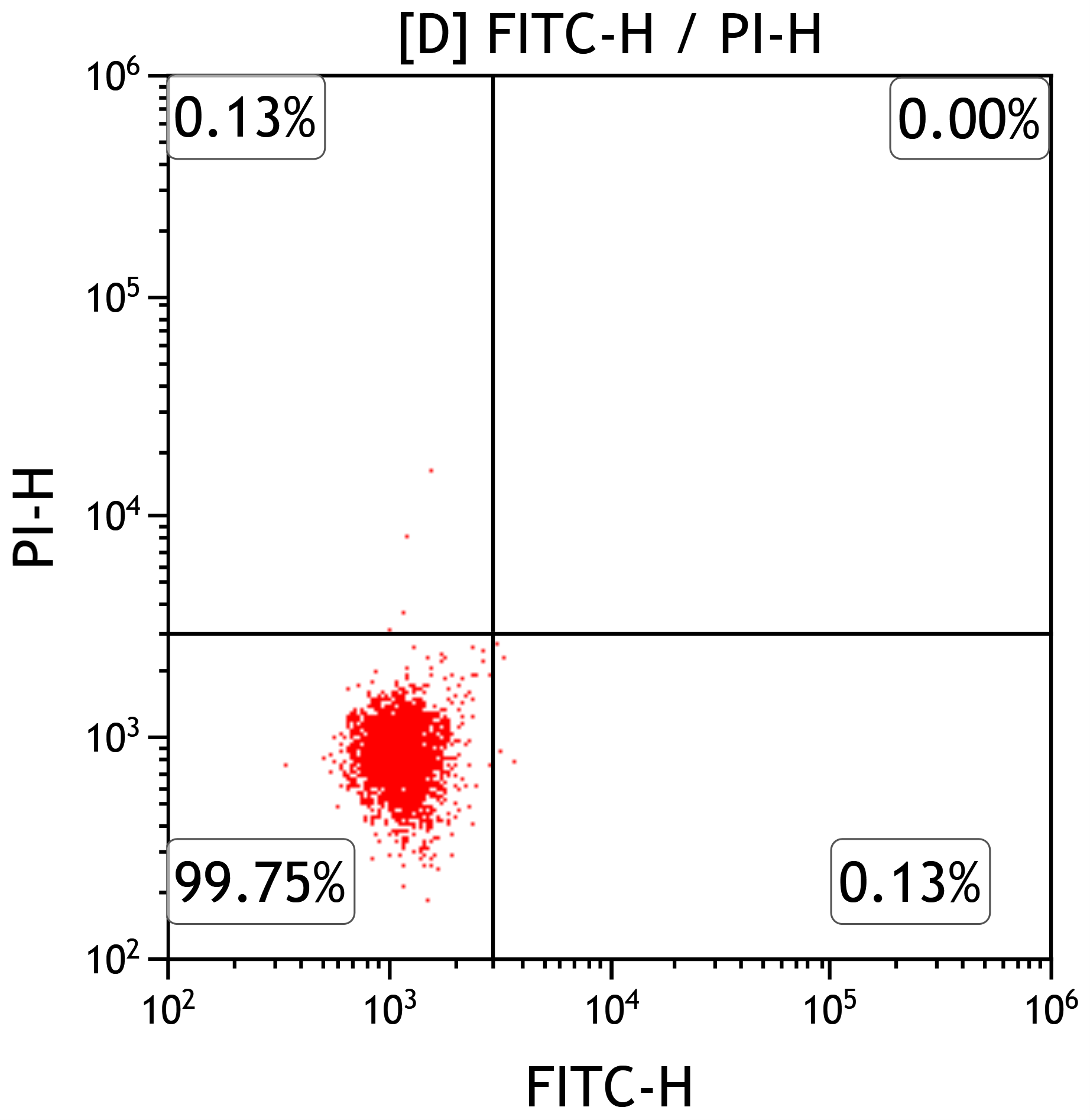

Supplement: Supplemental Material [file KBIE_A_2080412_SM3012.zip › Supplementary materials/apoptosis-FCM/FCM-Figure 7/miR-383-5p mimic+control-plasmid-2.png]

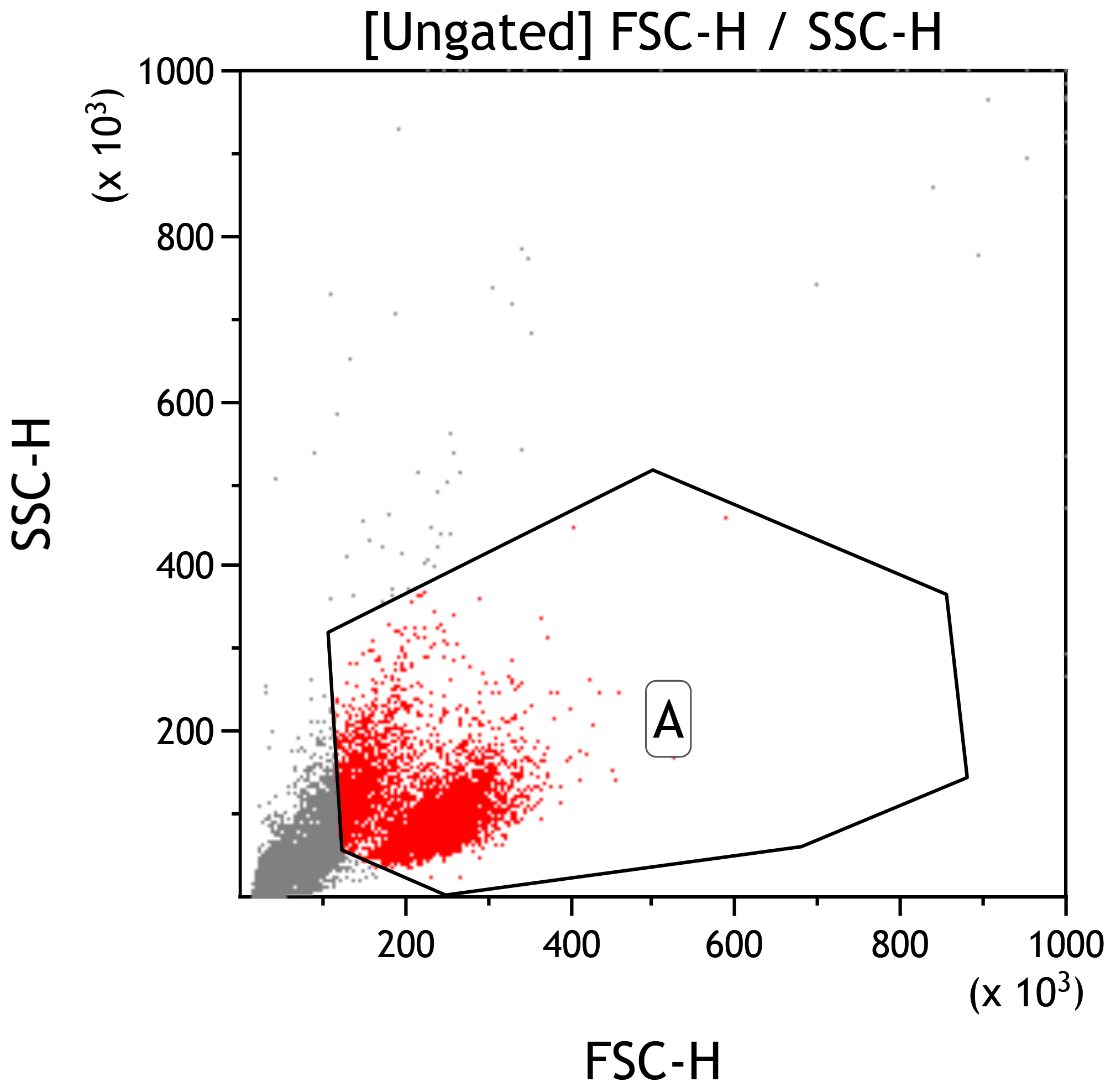

Supplement: Supplemental Material [file KBIE_A_2080412_SM3012.zip › Supplementary materials/apoptosis-FCM/FCM-Figure 7/miR-383-5p mimic-1.png]

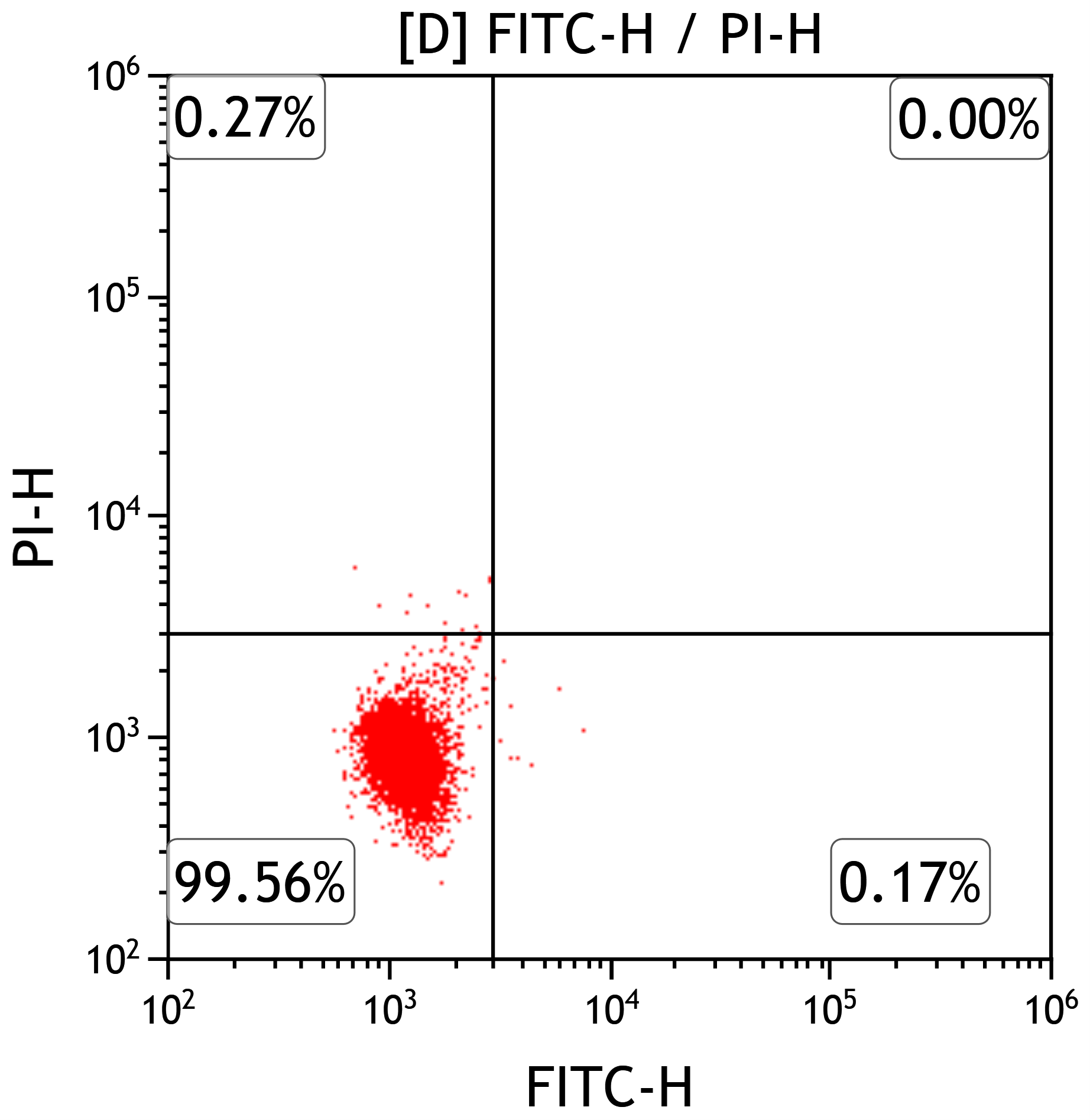

Supplement: Supplemental Material [file KBIE_A_2080412_SM3012.zip › Supplementary materials/apoptosis-FCM/FCM-Figure 7/miR-383-5p mimic-2.png]

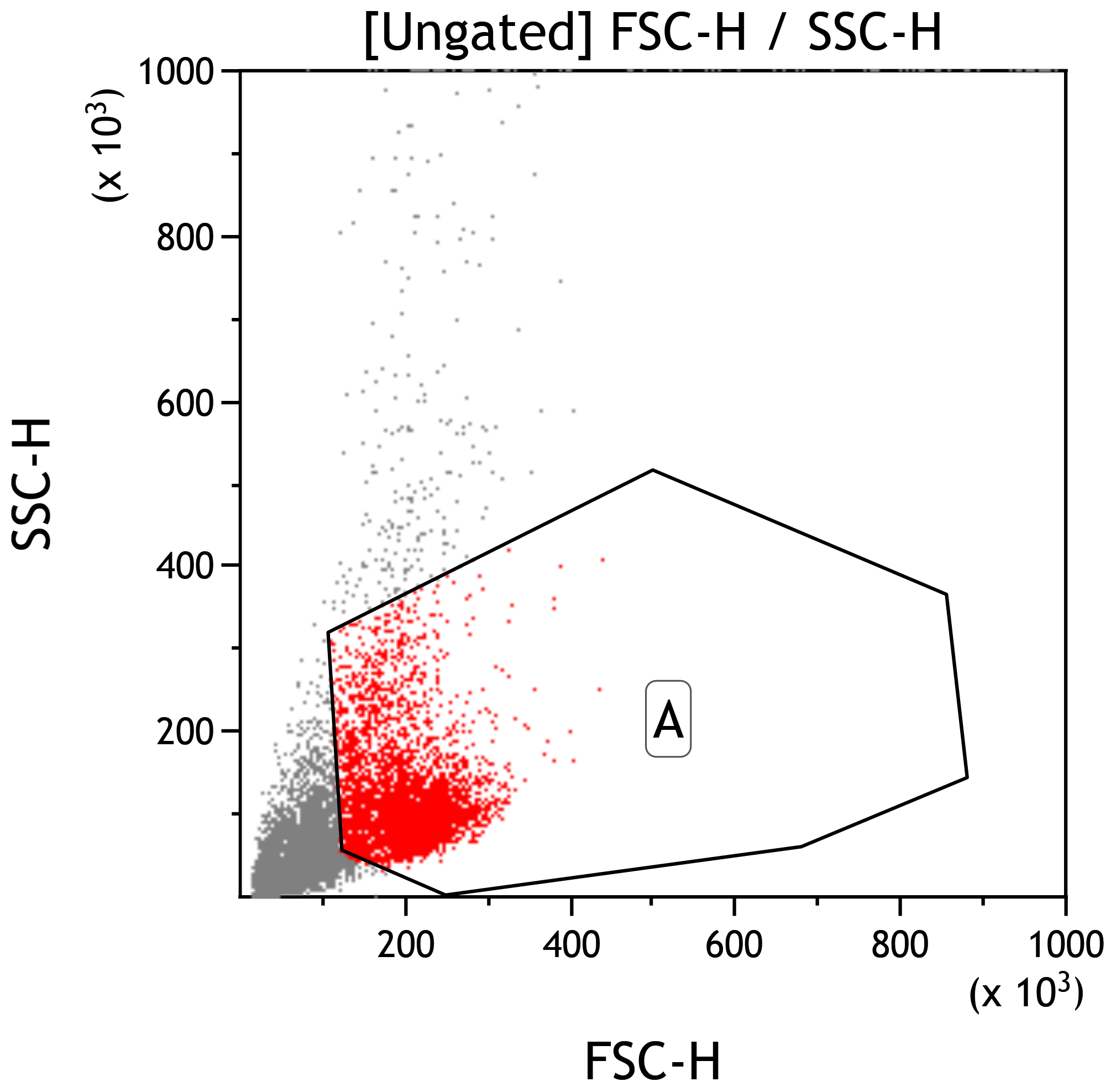

Supplement: Supplemental Material [file KBIE_A_2080412_SM3012.zip › Supplementary materials/apoptosis-FCM/FCM-Figure 7/mimic control-1.png]

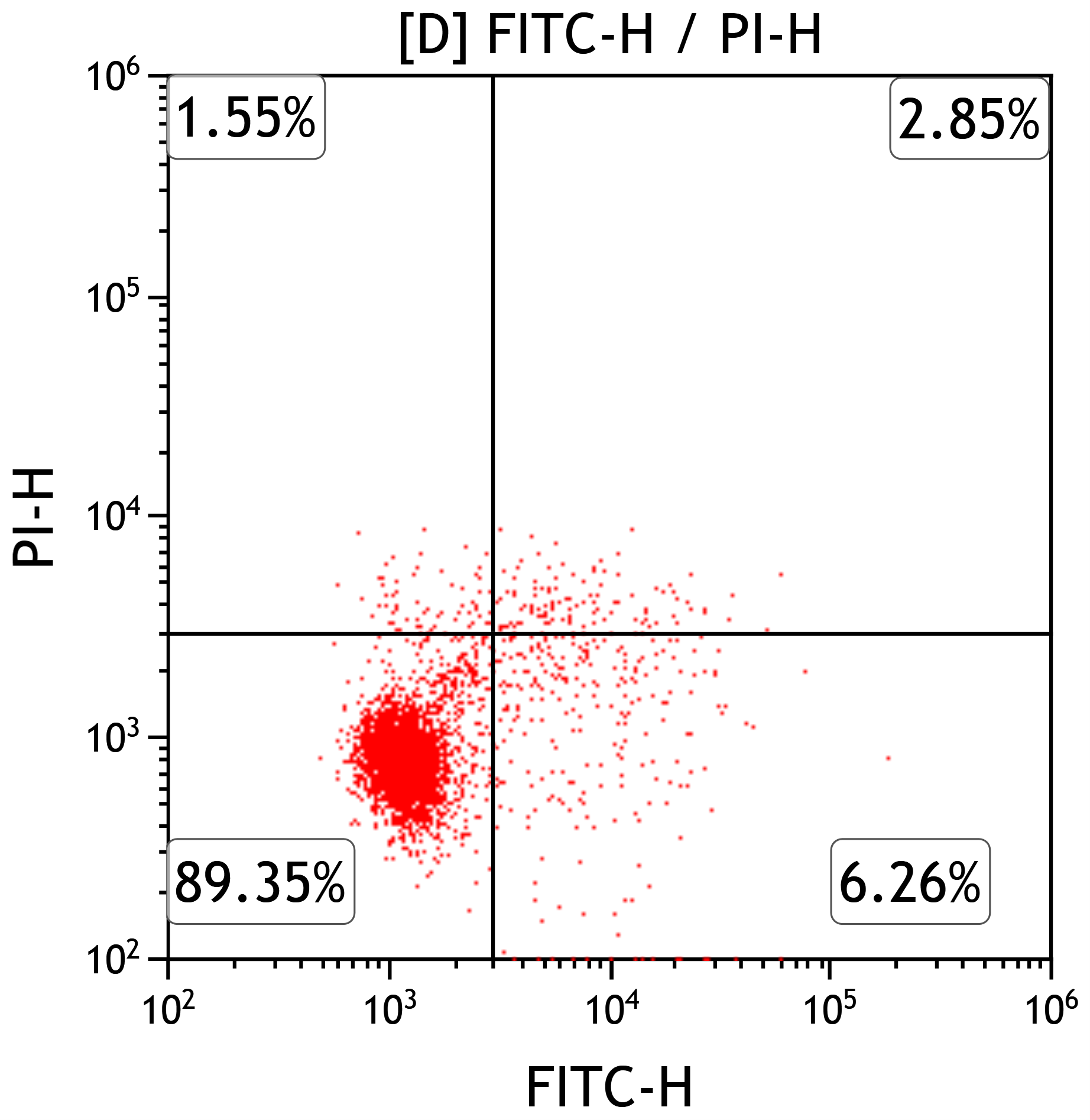

Supplement: Supplemental Material [file KBIE_A_2080412_SM3012.zip › Supplementary materials/apoptosis-FCM/FCM-Figure 7/mimic control-2.png]
